# Combined genetic and epigenetic interferences with interferon signaling expose prostate cancer cells to viral infection

**DOI:** 10.18632/oncotarget.10313

**Published:** 2016-06-28

**Authors:** Oded Danziger, Ben Shai, Yosef Sabo, Eran Bacharach, Marcelo Ehrlich

**Affiliations:** ^1^ Department of Cell Research and Immunology, George S. Wise Faculty of Life Sciences, Tel Aviv University, Tel Aviv, Israel

**Keywords:** human metapneumovirus, epizootic hemorrhagic disease virus, epigenetic silencing, interferon, prostate cancer cells

## Abstract

Interferons (IFNs) induce anti-viral programs, regulate immune responses, and exert anti-proliferative effects. To escape anti-tumorigenic effects of IFNs, malignant cells attenuate JAK/STAT signaling and expression of IFN stimulated genes (ISGs). Such attenuation may enhance the susceptibility of tumor cells to oncolytic virotherapy. Here we studied genetic and epigenetic mechanisms of interference with JAK/STAT signaling and their contribution to susceptibility of prostate cancer cells to viral infection. Bioinformatics analysis of gene-expression in cohorts of prostate cancer patients revealed genetic and epigenetic interference with the IFN program. To correlate lack of IFN signaling and susceptibility to viral infection and oncolysis; we employed LNCaP prostate cancer cells as cellular model, and the human metapneumovirus and the epizootic hemorrhagic disease virus as infectious agents. In LNCaP cells, JAK1 is silenced by bi-allelic inactivating mutations and epigenetic silencing, which also silences ISGs. Chemical inhibition of epigenetic silencing partially restored IFN-sensitivity, induced low levels of expression of selected ISGs and attenuated, but failed to block, viral infection and oncolysis. Since viral infection was not blocked by epigenetic modifiers, and these compounds may independently-induce anti-tumor effects, we propose that epigenetic modifiers and virotherapy are compatible in treatment of prostate tumors defective in JAK1 expression and IFN signaling.

## INTRODUCTION

Prostate cancers are the most diagnosed type of cancer and the second-highest cause of cancer-related cell deaths among men in the USA [1]. Multiple different molecular mechanisms induce and support prostate tumorigenesis, including point mutations, chromosomal alterations such as translocations, duplications, and deletions [2–4]; and aberrant epigenetic silencing of tumor-suppressor genes [5, 6]. These genetic and epigenetic alterations regulate cell-autonomous aspects of the prostate cancer cell such as excessive proliferation, invasiveness, and evasion of the tumor from immune surveillance. A plethora of signal transduction pathways and alterations to these pathways have been implicated in prostate cancer [7], including tumor-induced modifications to the cellular response to interferons (IFNs). While IFNs exert anti-proliferative and immune-modulatory functions, alterations to IFN signaling may coordinately modify intrinsic characteristics of prostate tumors, their interactions with the immune system, and their susceptibility to viral infections.

Signals of type I IFNs (IFNα and IFNβ) are transduced via a sequence of steps, including: (i) binding of IFNs to their designated receptor (consisting of two chains called IFNAR1 and IFNAR2), (ii) activation of receptor-associated Janus kinases (JAKs) by phosphorylation, (iii) phosphorylation of IFNAR1 and IFNAR2 intracellular tails, and creation of docking sites for latent signal transducers and activators of transcription (STATs), (iv) phosphorylation and oligomerization of STATs, in a complex which proceeds to directly bind DNA and regulate gene expression [8].

Type I IFNs induce tumor suppressor genes [9, 10] and exert anti-proliferative effects in a subset of prostate cancer models [11]. However, as a single agent, type I IFNs show limited efficiency in treatment of advanced prostate cancer [12]. This scenario stimulated two lines of research: development of combination therapy employing IFN with additional agents [13–15] and dissection of the molecular basis for IFN resistance. Concerning the latter line of research, molecular mechanisms of resistance to IFN in prostate cancer cells include inactivating mutations [16] or epigenetic silencing [17] of the IFN-activated kinase JAK1. Notably, epigenetic modifiers (EpMs) targeting DNA methylation or histone deacetylation, have also been proposed as therapeutic agents for prostate cancer [6, 18–20]. Thus, understanding the epigenetic regulation of the IFN response in prostate cancer cells may be of importance for optimal utilization of either EpMs or IFN in prostate cancer therapy. Importantly, while resistance to IFN in a subset of prostate tumor types is predicted to reduce the therapeutic potential of this cytokine, it is also expected to enhance the prospects of usage of oncolytic virotherapy, as IFNs are crucial for the induction of efficient antiviral state in IFN-responding cells [21].

LNCaP cells were isolated from human metastatic prostate adenocarcinoma found in a lymph node [22] and are a widely employed model of hormone-sensitive prostate cancer cell line [23] that recapitulates many molecular aspects of androgen-sensitive prostate cancers [24]. Moreover, multiple recent studies have addressed changes to gene expression in LNCaP cells upon inhibition of DNA methyltransferases (DNMTs) and/or histone deacetylases (HDACs) with 5-aza-2′-deoxycytosine (5AC) and/or trichostatin A (TSA), respectively [18, 25, 26]. These studies identified enhanced expression of a number of tumor-suppressor genes following treatment with EpMs, reinforcing the idea that LNCaP cells are a good model system for the study of aberrant epigenetic regulation in prostate cancer. Epigenetic silencing was also proposed to abrogate JAK1 expression in LNCaP cells, rendering them IFN-insensitive [17].

The IFN-insensitivity of LNCaP cells most likely contributes to their susceptibility to viral infections in general, and oncolytic viruses in particular (e.g. [27–30]). In this study we aimed to explore the inter-connectivity of IFN-insensitivity, aberrant epigenetic regulation and susceptibility to viral infection by probing the interaction of LNCaP cells with non-cytolytic and cytolytic RNA viruses. As a non-cytolytic virus, we opted for the human metapneumovirus (hMPV), a respiratory pathogen and a member of the *Paramyxoviridae* family. In a recent study we have inserted a GFP expression cassette into the full-length hMPV genome, generating hMPV-GFP [31]. This modified virus is a sensitive reporter of productive infection in live cells. Notably, hMPV both elicits and is sensitive to IFN-mediated anti-viral response [32]. As a cytolytic virus, we chose a variant of the epizootic hemorrhagic disease virus (EHDV), an orbivirus that naturally infects ruminants and is transmitted by biting midges [33]. When infecting mammalian cells, EHDV induces apoptosis, necrosis, autophagy and cell stress [34]. Notably, orbiviruses are strong inducers of the innate immunity/IFN response [35, 36], possibly due to their dsRNA genome.

The variability in the genetic and epigenetic etiology of prostate cancers raises the enticing prospect of personalized combination of different forms of therapy, including EpMs and virotherapy. To study the contribution of epigenetic regulation to the expression of IFN-stimulated genes (ISGs) in cells defective in IFN signaling we first explored the molecular basis of the refractoriness of LNCaP prostate cancer cells to IFN. We show that in these cells, JAK1 is silenced by both bi-allelic inactivating mutations and by epigenetic silencing. In addition, we demonstrated that the latter mechanism also plays a role in the silencing of ISGs. Furthermore, abrogation of epigenetic silencing, partially restored IFN-sensitivity, induced low levels of expression of some ISGs and attenuated, but failed to block viral infection and virally-induced cell death. Since viral infection was not blocked and EpMs may independently-induce anti-tumor effects, we propose that treatments of IFN, EpMs, and viral infection are compatible with each other in the context of JAK1 minus prostate tumor cells.

## RESULTS

### JAK1 inactivating mutations are present in subtypes of prostate cancers and in LNCaP cells, and perturb IFN signaling

**Figure 1:**
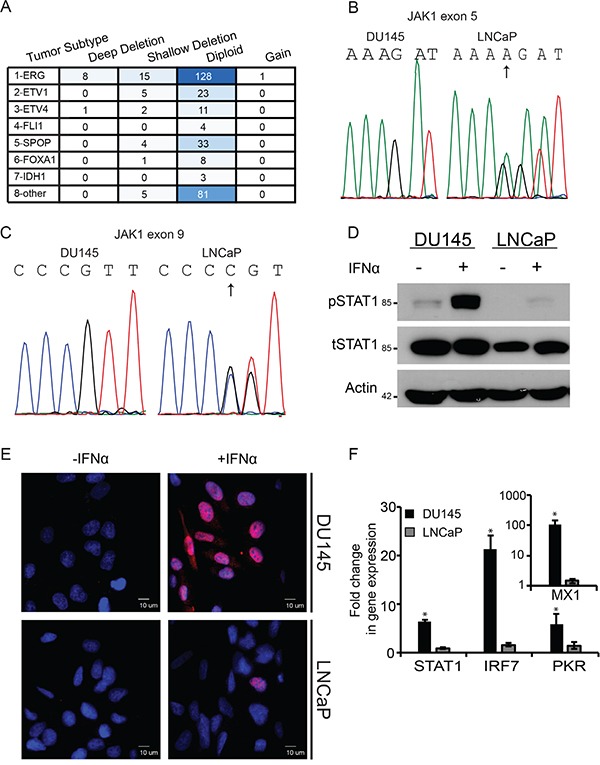
Deficient JAK1 expression characterizes a subset of prostate cancer patient samples and LNCaP cells, and correlates with lack of IFN signaling **A.** Table depicts the prevalence of different JAK1 genetic contents (ranging from deep deletion to gain) in different subtypes of prostate cancer; in a cohort of 333 patients (TCGA, cBioPortal, [37–39]. Different shades of blue are indicative of number of patient samples in category. **B.** Chromatograms of portion of exon 5 sequence of JAK1 in DU145 cells and LNCaP cells. The sequence that appears above the chromatogram of the LNCaP cells represents the mutant sequence; arrow marks the site of frameshift mutations (insertion of A). The chromatogram of LNCaP cells shows the mixture of wild-type and mutated sequences. **C.** Chromatograms of portion of exon 9 sequence of JAK1 in DU145 cells and LNCaP cells. The sequence that appears above the chromatogram of the LNCaP cells represents the mutant sequence; arrow marks the site of frameshift mutations (insertion of C). The chromatogram of LNCaP cells shows the mixture of wild-type and mutated sequences. **D.** IFN-mediated STAT1 phosphorylation is defective in LNCaP cells. DU145 and LNCaP cells were incubated with IFNα (200 U/ml, 4 h). Cells were extracted, protein lysates were separated by SDS-PAGE and immunoblotted with antibodies against the indicated proteins. Actin served as a loading control. **E.** Nuclear localization of pSTAT1 is impaired in LNCaP cells. LNCaP and DU145 cells grown on glass coverslips were treated with IFN as in (D). Cells were fixed, permeabilized and stained with DAPI (blue) and anti-pSTAT1/Alexa555-Goat-anti-Rabbit antibodies (red). Cells were imaged by immunofluorescence microscopy. Micrographs depict typical fields of the different cell lines prior to, or following IFNα stimulation. Bar. 10 μm. **F.** IFN-mediated induction of ISGs is defective in LNCaP cells. Graph depicts the fold change in gene expression in DU145 (black) and LNCaP (grey) cells, following IFNα stimulation (200U/ml, 4h) as measured by qRT-PCR. Expression of ISGs in independent experiments (n=4) was normalized to measured expression levels of housekeeping gene (GAPDH). Expression levels in unstimulated cells were taken as 1. *, p<0.005.

The complexity of regulation of IFN signaling in prostate cancer and the putative roles that ISGs exert in this malignancy, underscore the prospect of developing therapy combinations which alter ISG expression or exploit their lack of expression. To this end, there is a need to understand the interactions among mechanisms of epigenetic silencing, IFN signaling and susceptibility to viral infection in prostate cancer cells. Due to the central role played by JAK1 in IFN signaling, we first evaluated the prevalence of JAK1 mutations in prostate cancer by accessing the cBioPortal database [37, 38]. In the comprehensive TCGA cohort, composed of 333 patient samples [39], 3% of samples presented deep deletions in JAK1 (bi-allelic deletions in copy number analysis, CNA), while an additional 10 % of the samples presented shallow deletions (in one allele, Figure 1A). Further classification of this cohort into prostate cancer subtypes, revealed that 90 % of the JAK1 deep deletions occurred in the ‘ERG fusion’ subtype (p = 4.542e^−3^). These data show that genetic alterations to JAK1 are present in subtypes of prostate cancer cells. To study JAK1-defective prostate tumor cells, we opted for LNCaP cells as a model system; as Rossi et al., identified two heterozygous inactivating mutations in JAK1 gene [16]. In this study the authors failed to detect either JAK1 mRNA or its protein product in LNCaP and 22Rv-1 prostate cancer cell lines [16]. Thus, in normal growth conditions, the lack of expression of functional JAK1 in LNCaP cells should phenocopy prostate cancers with deep deletions in JAK1. To confirm the presence of these mutations in our batch of LNCaP cells, we extracted the genomic DNA from LNCaP and DU145 cells (the latter prostate cancer cell line served as a positive control since it is IFN-sensitive [17]). JAK1 specific primers were used to amplify, by PCR, exons 5 and 9 and the amplified DNA was sequenced. This analysis revealed the reported frameshift mutations in JAK1 [16] due to insertions of A and C in exons 5 and 9, respectively (Figure 1B-1C; arrows). We next examined the sequence of JAK1 transcripts, to evaluate the physical linkage (i.e., localization to the same allele) of the mutations. As shown by Dunn et al. [17] and described below, JAK1 expression in LNCaP cells is restricted by epigenetic silencing. To overcome this silencing LNCaP cells were treated by a combination of EpMs (5AC and TSA). In these treated cells, single JAK1 cDNA molecules amplified by RT-PCR were cloned, and the entire sequence encompassed between exon 5 and exon 9 was determined. These analyses revealed two different mutant JAK1 messages presenting either one of the missense mutations. The presence of two different JAK1 mRNA sequences in LNCaP cells demonstrates lack of linkage between the two inactivating mutations, leading to the conclusion that they are present on different alleles. Notably, 4 copies of chromosome 1 (where JAK1 is encoded) were shown to be present in LNCaP cells, by spectral karyotyping [40, 41]. Together, these data suggest that mutations in JAK1 occurred prior to chromosome duplication and demonstrate overlapping genetic and epigenetic molecular mechanisms of JAK1 inactivation in LNCaP cells.

To test the consequences of lack of expression of functional JAK1 in LNCaP cells on IFN signaling, we stimulated LNCaP and DU145 cells with IFNα (200U/ml, 4h), extracted total protein content from the cells and analyzed by immunoblotting total STAT1 (tSTAT1) and phosphorylated STAT1 (pSTAT1) levels. Figure 1D shows robust STAT1 phosphorylation in DU145 cells in response to IFNα, in contrast to only residual pSTAT1 levels in LNCaP cells. Similarly, when pSTAT1 levels were assayed by densitometry at 30 min post stimulation with 200U/ml IFNα, LNCaP cells exhibited only a 4.2±2.5 fold increase, while the increase in DU145 was much more robust (18±11 fold). Moreover, immunofluorescence analyses with antibodies against pSTAT1 in these cell lines, treated or untreated with IFNα, revealed widespread increase of pSTAT1 levels (red staining) and its nuclear localization, only in DU145 cells upon exposure to IFNα (Figure 1E). Furthermore, prominent transcriptional activation of selected ISGs (STAT1, IRF7, PKR and MX1, measured by qRT-PCR), was also observed only in DU145 cells, and not LNCaP cells (Figure 1F). To complement the qRT-PCR analyses, we measured the IFN response (200U/ml of IFNα for 4 h) in the above cells with a reporter gene (luciferase) under the control of multiple interferon sequence response elements (ISREs). These experiments (n=2) revealed that prior to IFN stimulation DU145 cells exhibit a 2.4±0.2 fold higher activity of the reporter than LNCaP cells. Moreover, and similarly to the qRT-PCR results, stimulation with IFN resulted in only residual (1.2±0.1 fold) activation in the latter cell line. Together, these results confirm the low sensitivity of LNCaP cells to IFN, which correlates with the presence JAK1 inactivating mutations.

### Epigenetic silencing of components of the IFN system in prostate tumors and in LNCaP cells

**Figure 2:**
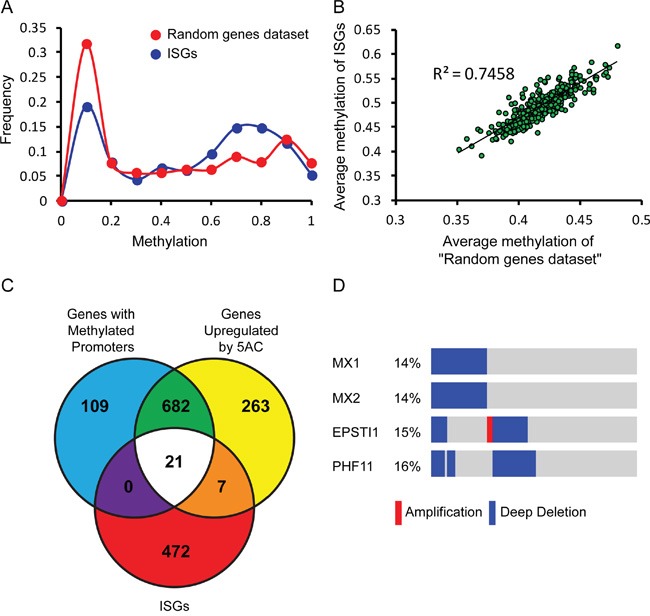
Genetic and epigenetic mechanisms contribute to negative regulation of ISG expression in prostate cancer patient samples and LNCaP cells **A.** Graph depicts the frequency of β values (methylation) of 500 randomly selected human genes (red) or 500 ISGs (blue, [43]) in prostate cancer patient samples (TCGA, cBioPortal, [37–39]). **B.** Graph depicts a per-patient correlation between the average β value of the 500 randomly selected human genes and the average β value of the 500 ISGs (same data sets as in (A)). **C.** Venn diagram depicts intersections among gene lists of: “genes with methylated promoters” in LNCaP cells, “genes up-regulated by 5AC treatment” in LNCaP cells [26], and 500 ISGs [43]. **D.** Graphical depiction of prostate cancer patient samples (TCGA, cBioPortal, [37–39] with deep deletion (blue), amplification (red), or no alteration (gray) of the genetic content of the indicated genes. The percentages of these alterations in the cohort are shown on the left. Patient samples are distributed along the bars; mutations that appear in the same sample are aligned above each other.

In addition to IFN-insensitivity, negative regulation of gene expression due to epigenetic silencing has been proposed as a hallmark of LNCaP cells [17, 18, 25, 26, 42]. To explore the possible connection between epigenetic silencing and defective IFN response in prostate cancer patients, we initially estimated the extent of DNA methylation of ISGs in patient samples. For this, we analyzed the distribution of β values ([39], representing methylation levels as measured with the Illumina HM450 BeadChip) of 500 ISGs [43] and of 500 randomly selected human genes (‘random genes dataset’). Such analysis revealed a decrease in the portion of ISGs relative to the random gene dataset at low β values (0<β<0.2), and an increase in the portion of ISGs at β values ranging between 0.5 and 0.8 (Figure 2A). The increase in β values of the ISGs is indicative of a higher tendency of methylation of these genes, and suggests that the expression of these genes is negatively regulated by epigenetics. Interestingly, a per-patient correlation of the average β value of the ‘random gene data set’ and of the ISG data set revealed a positive correlation (R squared=0.75) between both values (Figure 2B); suggesting that tumors which show increased tendency for DNA methylation, also show increased methylation of ISGs. To estimate the extent of epigenetic silencing of ISGs in LNCaP cells, we compared a list of 500 ISGs [43], with LNCaP-derived gene lists, consisting of 973 genes presenting modified expression following treatment with the DNA methyl transferase inhibitor 5AC and 812 genes with methylated promoters [26]. Such analysis revealed a subgroup of 21 genes common to all three categories (Figure 2C), suggesting the epigenetic suppression of ISG expression in LNCaP cells. To analyze if genetic mechanisms are involved in the inactivation of genes belonging to this group in the clinical prostate cancer samples we accessed cBioPortal. This analysis revealed deep deletions of a subset of these genes (Figure 2D, blue). For example, the ISGs MX1 and MX2 show deep co-deletions in 14 % of prostate cancer patients (Figure 2D). A probable explanation for the high prevalence of their co-deletion is that both MX1 and MX2 localize to the region between TMPRSS2 and ERG (chromosome 21). This region is deleted upon fusion of TMPRS22 and ERG, which is commonly found in prostate cancer [44]. Similarly, the genes EPSTI1 and PHF11 (also part of the 21 gene group) localize to chromosome 13 and are co-deleted in ~15% of prostate cancer patients (Figure 2D). Together, these data suggest that the interference with expression of ISGs in prostate cancer cells, via genetic and/or epigenetic mechanisms, may be a general feature of this malignancy, and may contribute to both oncogenesis and sensitization of prostate cancer cells to viral infection. These data also highlight LNCaP cells as a good model for prostate tumors, in which combinations of genetic and epigenetics alterations inactivate components of the IFN system.

**Figure 3:**
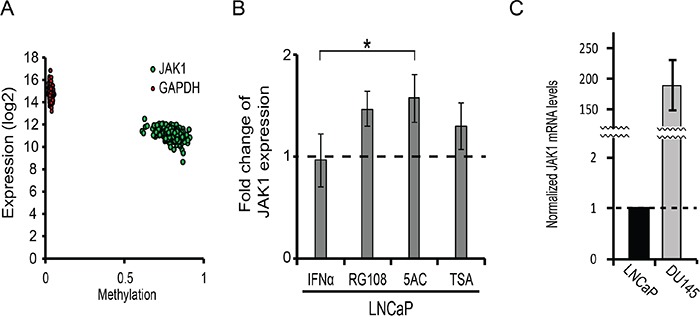
JAK1 is epigenetically regulated in prostate cancer patient samples and LNCaP cells **A.** Graph depicts the per-patient correlation between the β values (methylation) and the expression (mRNA) of JAK1 (green) or GAPDH (red). **B.** Graph depicts the relative mRNA levels of JAK1 (normalized to GAPDH mRNA, average ± SD, n=3, p<0.05) in LNCaP cells, stimulated or not with IFNα, and treated or not with the indicated EpMs. The expression levels in untreated and unstimulated cells in each independent measurement were taken as 1 (dashed line). **C.** Graph depicts the normalized mRNA levels of JAK1 (relative to GAPDH mRNA) in LNCaP and DU145 cells (average ±SD, n=3, ***, p<0.005).

Since epigenetic silencing of JAK1 was reported in prostate cancer cell lines [17, 45], we next analyzed the extent of methylation of JAK1 in patient-derived samples of the TCGA cohort [39], and correlated this methylation with JAK1 expression levels (Figure 3A). As control, a similar analysis was performed with the housekeeping gene GAPDH. JAK1 exhibited higher levels of methylation than GADPH (β values of 0.78 ± 0.05 for JAK1, as opposed to 0.033 ± 0.006 for GAPDH) and stricter negative correlation between methylation and expression (Pearson's correlation of −0.5 for JAK1, as opposed to −0.2 for GAPDH). These data suggest that epigenetic modifications contribute to JAK1 down regulation in prostate cancer patient samples. To probe for the epigenetic silencing of JAK1 in the LNCaP model, we opted to treat these cells with different epigenetic modifying agents that target either DNA methylation or histone de-acetylation. Specifically, we employed 5AC, RG108 (a specific DNMT1 inhibitor, [46]) or TSA, and measured JAK1 expression by qRT-PCR. Treatment of LNCaP cells with the EpMs resulted in low but measurable increases in JAK1 mRNA (Figure 3B), confirming the contribution of epigenetic modifications to reduced expression of JAK1 message in this cell line. The low expression of JAK1 mRNA (normalized to GAPDH) in LNCaP cells, was further apparent upon the comparison of this normalized expression to the expression observed in DU145 cells (~ 180 fold higher in DU145, Figure 3C).

**Figure 4:**
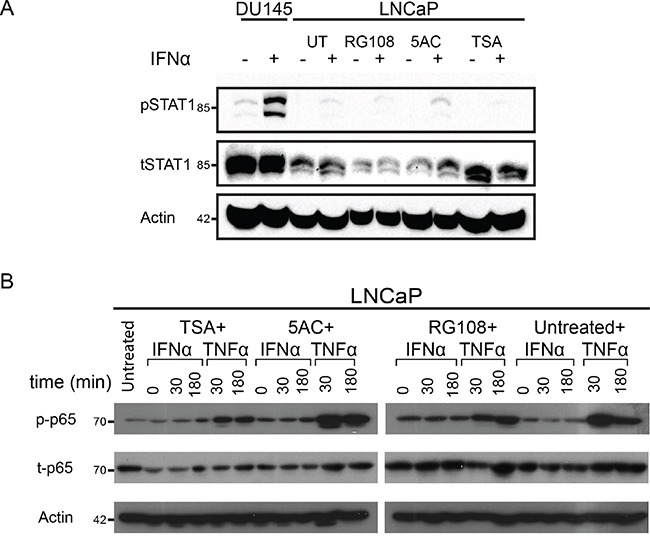
Treatment of LNCaP cells with EpMs fails to increase canonical and non-canonical IFN response **A.** LNCaP cells were pre-treated, or not, for 24 h with the indicated EpMs, and stimulated, or not, with IFNα (200 U/ml, 4 h). Cell lysates were separated by SDS-PAGE, blotted and probed with antibodies against the indicated proteins. Untreated DU145 cells (stimulated, or not, with IFNα) served as a positive control. In the shown representative experiment, densitometry of tSTAT1/actin yielded the following ratios (from left to right): 0.82, 0.72, 0.5, 0.5, 0.3, 0.3, 0.36, 0.4, 0.63, 0.5. B- LNCaP cells were pre-treated as in (A) and stimulated, or not, with either IFNα (200 U/ml) or TNFα (10 ng/ml) for the indicated time periods. Immunoblots, prepared as in (A), were probed with antibodies against the indicated proteins. In both (A) and (B), actin served as a loading control.

Since IFN-stimulation still induced a residual phosphorylation of STAT1 in LNCaP cells (Figure 1), it was not clear if abrogation of epigenetic silencing may potentiate IFN-induced ISG expression and/or reduce the infectibility of LNCaP cells. Moreover, in addition to the JAK1-STAT1 axis, IFN was shown to activate the NFκB transcription factor in the absence of JAK1 [47]; such activation has the potential to regulate a plethora of genes including multiple ISGs (see Supplementary Table 2). We initially probed for pSTAT1 levels under different experimental conditions of EpM treatments and/or IFN stimulation. In LNCaP cells, IFN induced only residual levels of pSTAT1, in either untreated cells, or cells treated with EpMs (Figure 4A), indicating that treatment of the cells with EpMs does not restore robust IFN signaling. Densitometry measurements of the ratio between tSTAT1 and actin (Figure 4A) revealed greater tSTAT1 content in DU145 cells as compared to LNCaP cells and no significant increase in tSTAT1 content of the latter cells, upon EpM treatments. We next probed for phosphorylation of the NFκB p65 subunit (p-p65), as indicative of NFκB activation, in LNCaP cells treated or untreated with EpMs and stimulated or not with IFNα. Stimulation of LNCaP cells with tumor necrosis factor α (TNFα) served as a positive control for NFκB activation. While TNFα induced a robust p-p65 signal in LNCaP cells, irrespective of EpMs, no increase in p-p65 was observed upon IFNα stimulation in either untreated cells, or cells treated with EpMs (Figure 4B). Altogether, treatment of LNCaP cells with EpMs failed to increase the IFNα-induced residual activation of the canonical (STAT1) pathway, or to induce the non-canonical (NFκB) pathway.

### EpMs reduce, but do not block, infectibility of LNCaP cells

**Figure 5:**
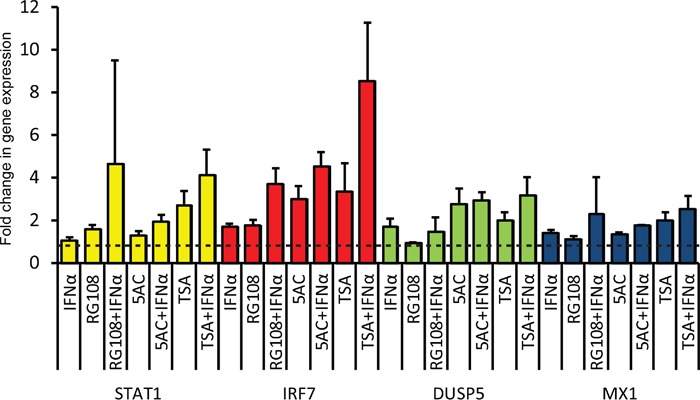
Treatment of LNCaP cells with EpMs and/or IFN results in minimal increases in expression of ISGs LNCaP cells were pre-treated as in Figure 4, and stimulated, or not, with IFN (in presence or absence of EpMs) for 4 h prior to RNA extraction. Graph depicts average ± SEM (n= 4) of qRT-PCR measurements of the mRNA levels of indicated genes, normalized to GAPDH mRNA. Dashed lines represents the level of normalized expression in untreated (no IFN, no EpMs) cells, taken as 1.

To probe for the effect of EpMs, in combination or not with IFN, on the expression of a subset of ISGs in LNCaP cells, we measured by qRT-PCR the relative expression levels of STAT1 and IRF7 (ISGs that transduce the IFN signal [48]), and MX1 and DUSP5 (ISGs that play roles in innate immunity and also show anti-oncogenic activities in prostate cancer [49, 50]). As a single agent, addition of IFN resulted in minimal increases (less than 2 fold) in each of the tested ISGs (Figure 5). Similarly, the increases in expression observed with EpMs (as single agents) were restricted to a maximum of 3 fold. For all of the combined treatments (IFN plus each of the EpMs) the observed average fold change in gene expression was higher than for single agents. This suggests that abrogation of epigenetic silencing increased responsiveness to IFN to some extent. The restricted increases in expression of ISGs observed in LNCaP cells (even upon combined treatment of EpMs and IFN) are in sharp contrast with the increases observed in IFN-stimulated DU145 cells (MX1,~100 fold; IRF7, ~20 fold, Figure 1F). Together, our data suggest the centrality of the lack of functional JAK1 in restraining the IFN responsiveness of LNCaP cells.

**Figure 6:**
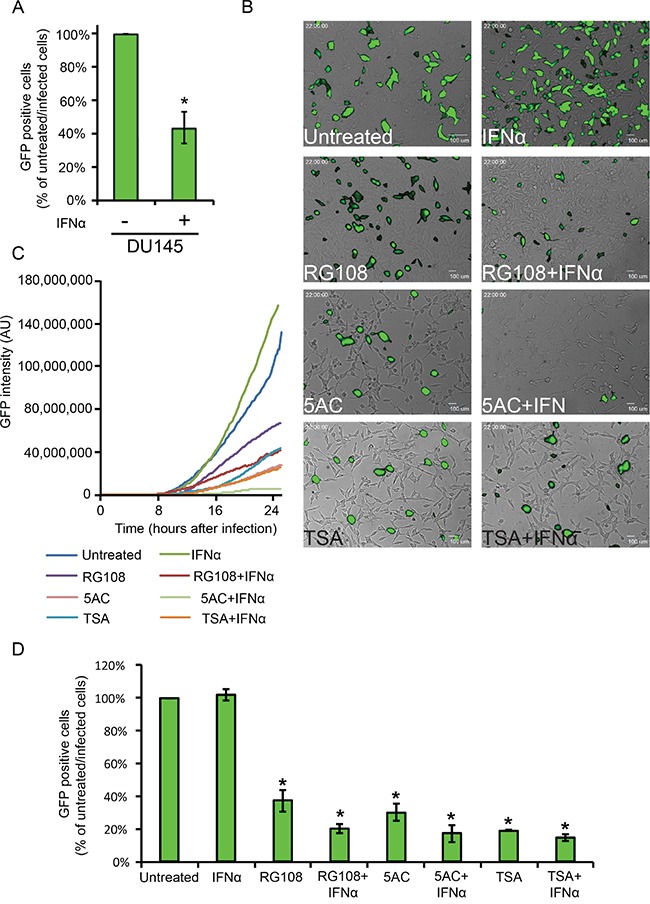
EpMs reduce hMPV-GFP infection in LNCaP cells **A.** DU145 cells were pretreated, or not, with IFNα (200 U/ml, 4h) and infected for 24 h with hMPV-GFP (0.5 m.o.i), prior to FACS analyses of percentage of infected cells. Graph depicts the average ± SD percentage of hMPV-GFP infected cells, where the percentage of infected cells without IFNα treatment is taken as 100%. **B.** Micrographs depict the 22 h time-point of the live-cell microscopy time-lapse sequences of hMPV-GFP-infected LNCaP cultures, under the indicated conditions of EpM/IFNα treatments. LNCaP cells were pre-treated as in Figure 4 and infected with hMPV-GFP (24 h, in medium conditions identical to pre-treatment). **C.** Graph depicts the accumulation of GFP signal (normalized to the number of cells in the field) of the hMPV-GFP infections visualized by live-cell microscopy. **D.** LNCaP cells were pretreated, infected and analyzed as above. Graph depicts the average ± SD percentage of hMPV-GFP infected in the indicated conditions, where the percentage of infected cells without treatments was taken as 100%. *, p<0.005 (compared to untreated/infected cells).

If the modest, yet consistent, increase in the measured expression of the subset of ISGs following treatment with EpMs (in absence or presence of IFN) is indicative of changes to expression of the ISGome as a whole, this may suffice to mount an antiviral state in LNCaP cells. To directly test if the above treatments alter the susceptibility of LNCaP cells to viral infections we initially employed a human metapneumovirus engineered to express GFP in infected cells (hMPV-GFP, [31]). In the conditions employed here, hMPV-GFP is not lytic, and GFP expression reports on early stages of infection in single live cells. To examine the susceptibility of hMPV-GFP to IFN-mediated restriction of infection, we employed the IFN-responsive DU145 cell line. As shown in Figure 6A, IFN significantly reduced hMPV-GFP infection in DU145 cells. To characterize hMPV-GFP infection in LNCaP cells, we initially opted to visualize infected LNCaP cultures by live-cell microscopy. Specifically, semi-confluent LNCaP cells, pre-treated or not with EpMs (24 h, RG108, 5AC or TSA) and/or IFNα (4 h, before infection), were infected with hMPV-GFP (m.o.i 0.5) and imaged by phase contrast and fluorescence microscopy for an additional 24 h (in same conditions as pre-treatment: ± EpMs, ± IFN). In untreated cells, GFP signals were first observed at ~ 9 h post infection, after which they continuously accumulated throughout the time-lapse sequence (Movie S1, representative picture at 22 h hpi, Figure 6B; Figure 6C, blue line). IFNα-treated LNCaP cells showed very similar pattern of GFP accumulation (Figure 6B; Figure 6C, green line), indicating a lack of inhibitory effect of IFN as a single agent. In contrast, EpMs reduced accumulation of GFP signal as single agents (Figure 6B and Figure 6C, RG108, purple line; 5AC, pink line; TSA, cyan line). Combination of EpMs with IFN resulted in further inhibition of GFP accumulation (Figure 6B and Figure 6C, RG108+IFN, maroon line; 5AC+IFN, light green line, TSA+IFN, orange line). As microscopy measurements follow single fields of cells, we complemented these measurements by analyzing the percentage of GFP positive cells (at 24 h post-infection) by fluorescent activated cell sorting (FACS, Figure 6D). The FACS analyses confirmed the tendencies observed by live-cell microscopy, where considerable inhibition of hMPV-GFP infection was observed with EpMs as single agents, and an increment to these inhibitions was observed upon co-treatment of EpMs and IFNα. Together, these data support the notion that epigenetic silencing of ISGs contribute to the hypersensitivity of LNCaP cells towards infection and that reversal of this silencing reduces the infectibility of LNCaP cells and partially sensitizes these cells to protective effects of IFN.

**Figure 7:**
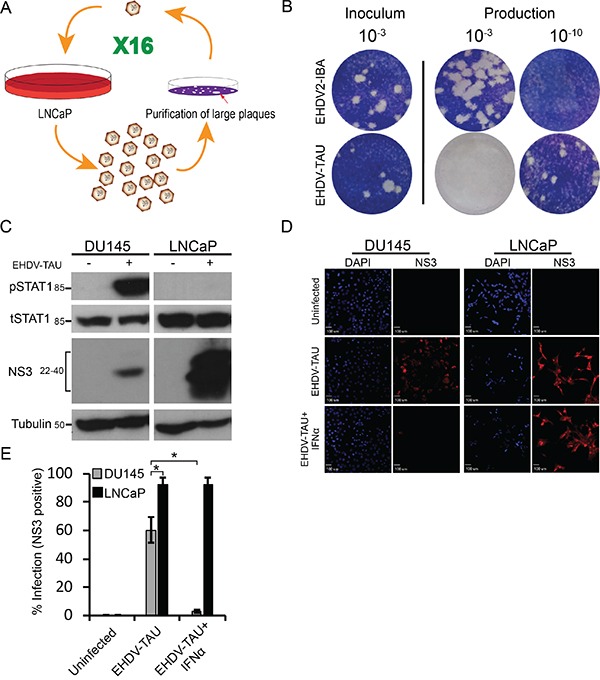
Serially passaged EHDV (EHDV-TAU) differentially infects LNCaP and DU145 cells **A.** Schematic depiction of selection procedure. The single virion represents a selected clonal, plaque-purified virus; whereas the multiple virions represent diverse virus populations (quasispecies). **B.** Plaque assay analysis of the fold increase in titer for EHDV-TAU, compared to EHDV2-IBA, in LNCaP cells. Panel depicts typical images of the plaque assays; the dilution employed appears above the respective wells. Left inoculum (10^−3^ dilution); right, dilutions (10^−3^ and 10^−10^) of the virions (of the indicated viruses) produced in LNCaP cells (60 h infection) **C.** Immunoblot analysis of NS3 production and STAT1 phosphorylation in EHDV-TAU-infected cells. Lysates (100 μg protein) of DU145 or LNCaP cells, infected or not with EHDV-TAU (0.05 pfu/cell, 45 h) were separated by SDS-PAGE, blotted and probed with antibodies against the indicated proteins. α-tubulin was used as a loading control. D-E Exogenous addition of IFNα blocks EHDV-TAU infection in DU145 cells, but not in LNCaP cells. **D.** Panels depict typical fields of DU145 and LNCaP cells, stained for DAPI (blue, left panels) and NS3 (red, right panels) under the indicated conditions: uninfected, EHDV-TAU-infected (45 hpi, 0.05 pfu/cell), or EHDV-TAU infected (45 hpi, 0.05 pfu/cell) treated with IFNα (200U/ml, 45 h). Bar, 100 μm. **E.** Graph depicts the mean ± SE percentage of NS3 positive cells. Quantification of percentage NS3 positive cells was from multiple (n=5) randomly selected fields, imaged under the same conditions as in (D). *, p<0.05.

Naturally oncolytic viruses are tumor virotherapy agents characterized by replication competence and intrinsic ability to selectively infect and kill cancer cells [21, 51]. Amongst the different naturally oncolytic viruses under current experimentation are the mammalian reovirus [52–54], a natural virus of humans that does not cause disease; and veterinary viruses such as the Newcastle disease virus (NDV, [55]), vesicular stomatitis virus (VSV, [56, 57]) and myxoma virus (MYXV, [58]). Here, we decided to pursue the attractive possibility of combining the advantages of both reoviruses and veterinary viruses. To this end, we chose the Ibaraki strain of the Epizootic Hemorrhagic Disease virus (EHDV2-IBA), as we previously showed that when infecting mammalian cells, EHDV2-IBA is cytolytic and induces apoptosis, necroptosis, autophagy and cell stress [34]. We hypothesized that serial passaging of EHDV2-IBA in LNCaP cells (schematically depicted in Figure 7A) may optimize its infection abilities of human cancer cells. Indeed, infection of LNCaP cells with the serially passaged virus (denominated ‘EHDV-TAU’), augmented by 6 orders of magnitude the fold increase in titer of infected cultures (60 h infection, m.o.i 0.05, Figure 7B). To probe for selectivity of EHDV-TAU to cancer cells, we compared its infection in LNCaP cells and in immortalized, non-transformed prostate cells (EP cells; [59]). The synthesis of non-structural proteins is an indicator of productive infection. Infection of LNCaP cells with EHDV-TAU resulted in readily detectable levels of the non-structural protein 3 (NS3), while no NS3 was detected in EP-infected cultures (Supplementary Figure 1A). In accord with a differential susceptibility towards EHDV-TAU infection, crystal-violet staining (45 hpi) of LNCaP- and EP- cell cultures infected (m.o.i of 0.05), or not, with EHDV-TAU, revealed extensive reduction in staining in the infected LNCaP culture (interpreted as extensive cell death), while no reduction in staining was observed in infected EP culture (Supplementary Figure 1B). We next compared the infection of EHDV-TAU in LNCaP cells and in DU145 cells. The synthesis of non-structural proteins is an indicator of productive infection. As seen in Figure 7C, infection of LNCaP cells with EHDV-TAU resulted in ample expression of NS3, while much lesser levels of NS3 were detected in DU145 infected cultures. To test if EHDV-TAU elicits an IFN response in DU145 or LNCaP infected cells, we measured the amount of pSTAT1 in presence or absence of EHDV-TAU infection. EHDV-TAU induced readily detectable levels of pSTAT1 in DU145 cells, while no such signal was observed in the infected LNCaP cell culture (Figure 7C). To examine if IFN addition restricts EHDV-TAU infection, we infected DU145 or LNCaP cells, in presence or absence of IFNα, and measured the percentage of NS3-positive cells by immunofluorescence microscopy. As shown in Figure 7D-7E, addition of IFNα abrogated the NS3 signal from EHDV-TAU-infected DU145 cultures, while having negligible effects on the infection of LNCaP cells.

**Figure 8:**
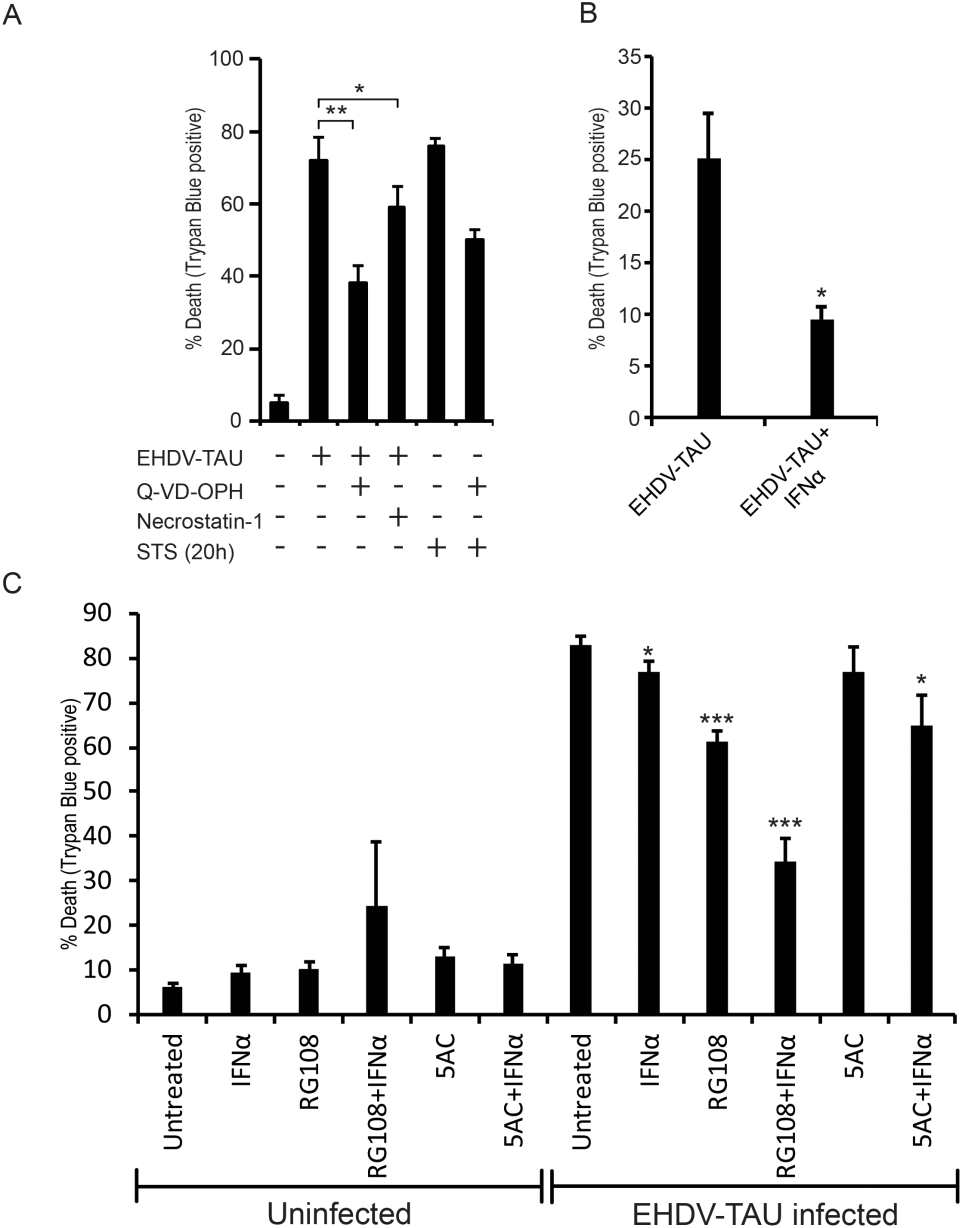
EHDV-TAU induces apoptotic and non-apoptotic cell death, which is only partially inhibited by EpMs and IFNα **A.** LNCaP cells, infected (+) or not (−) with EHDV-TAU (0.05 pfu/cell, 45 h) and treated (+) or not (−) with Q-VD-OPH (20 μM, 45 h in infection, or 20 h with STS treatment), Necrostatin-1 (75 μM, 45 h) or STS (positive control of apoptosis induction, 1 μM, 20 h) were analyzed by trypan blue exclusion assay to determine percentages of dead cells. Graph depicts mean ± SE (n= 3) of the percentage of cell death under the indicated conditions. *, p<0.05; ** p<0.005. **B.** DU145 cells, treated or not with IFNα (200 U/ml) were infected with EHDV-TAU (m.o.i. 0.05, 45 h) and cell death was analyzed as in (A). Graph depicts mean ± SE (n= 3) of percentages of cell death under the indicated conditions. *, p<0.05. **C.** LNCaP cells, infected or not with EHDV-TAU (0.05 pfu/cell, 45 h) and treated, or not, with IFNα or the indicated EpMs were analyzed for cell death as in (A). Graph depicts mean ± SE (n= 5) of percentage of cell death under the indicated conditions. *, p<0.05; ***, p<0.0005.

Infection of LNCaP cells with EHDV-TAU resulted in a progressive accumulation of dead cells, peaking at ~ 72 h (data not shown). To probe for the mechanism of EHDV-TAU-induced death of LNCaP cells, we treated infected cultures with either Q-VD-OPH (pan-caspase inhibitor) or Necrostatin-1 (necroptosis inhibitor, [60]), and observed a partial reduction in killing by EHDV-TAU, with each of the inhibitors (Figure 8A). These data suggest that EHDV-TAU kills LNCaP cells by both apoptotic and non-apoptotic pathways. We next probed for the effects of EpMs and/or IFN on the ability of EHDV-TAU to kill LNCaP cells. Preliminary experiments demonstrated that the time-frame of the experiment (84 to 96 h, including pre-treatments and infection), impeded the use of TSA, as it was toxic to LNCaP cells at longer incubation periods (in contrast to 48 h incubation, see Supplementary Movie 7). Addition of IFN alone was sufficient to significantly protect DU145 cells (Figure 8B). In sharp contrast, only a minimal reduction in EHDV-TAU-induced cell death of LNCaP cells was observed upon IFNα addition (Figure 8C). RG108 alone presented a partial protection of LNCaP cells, and this protection was increased upon combination treatment with RG108 and IFNα (Figure 8C). 5AC partially sensitized cells to the protective effect of IFNα, as the combination of both agents reduced infection to a greater and significant degree, as compared to IFNα or 5AC alone. Yet, the combined treatment of IFN and 5AC offered a lesser protection to the cells from virus-induced death, compared to the combined treatment of IFNα and RG108. Together, these data demonstrate a mild anti-viral response elicited by EpMs in LNCaP cells, and the increase of such response in presence of IFNα. However, even the most effective anti-viral drug combination (IFN and RG108) did not block virus-induced cell death but rather reduced it to half of the level observed in untreated, infected cells. The limited extent of the anti-viral effects of EpMs, IFNα or their combination, suggests that genetic defects in JAK1/STAT1 signaling may play a prominent role in determining the susceptibility of a subset of prostate cancer cells to virotherapy, even under conditions of combined treatment with EpMs.

## DISCUSSION

In the present study we employed the abundantly researched hormone-dependent LNCaP prostate cancer cell line and two different RNA viruses, hMPV and EHDV, to explore the functional interaction between two features of prostate cancers: aberrant epigenetic regulation of gene expression [6, 17–20, 25, 26, 42] and defects in IFN signaling or expression of ISGs [16, 17, 45, 61]]. Understanding the functional interactions between IFN signaling and epigenetic silencing in tumor cells is important because of the therapeutic potential of EpMs, IFN and oncolytic viruses in prostate cancer therapy [14, 15, 61–65].

LNCaP cells are characterized by IFN insensitivity and different molecular mechanisms of dampening of JAK1 expression. Our data show that negative regulation of JAK1 expression in LNCaP cells occurs through both epigenetic silencing and frameshift mutations. Thus, while these cells are tetraploid relative to chromosome 1 (where the JAK1 gene localizes; [40, 66]), all alleles present different frameshift point mutations (in exon 5 or 9) leading to premature stop codons and inactive protein products, as the truncated JAK1 terminates before the kinase domains. In addition to impeding the synthesis of functional JAK1, these mutations expose the mutated JAK1 message to nonsense-mediated decay [16], further reducing JAK1 mRNA levels. The fact that point mutations were observed in both JAK1 alleles suggests that LNCaP may be prone to such genetic aberrations. Notably, LNCaP cells present a deep deletion in chromosome 2 (p16) [66], spanning MSH6 locus. Mutations in MSH6 result in defects in DNA mismatch repair and increased risk for several malignancies as was demonstrated for Lynch syndrome [67]. The notion of epigenetic silencing of JAK1 in LNCaP cells is supported by the increase in JAK1 mRNA upon treatment with EpMs (RG108, 5AC, TSA), similarly to other published reports [17, 45]. As no functional JAK1 is encoded in LNCaP cells, treatments with EpMs failed to add to the residual increase in pSTAT1 upon IFN stimulation (Figure 4). A possible explanation for the observed residual activation of STAT1 in LNCaP cells is JAK1-independent STAT1 phosphorylation, similarly to what was observed upon overexpression of Tyk2 in U937 monocytes [68].

Even though EpM treatments failed to increase pSTAT1 levels following IFN stimuli, the interference with epigenetic silencing of ISGs contributed to the restriction of hMPV-GFP infection, but fell short of complete inhibition of such infection. If reproducible *in vivo*, it would suggest the advantage of concomitantly challenging ‘LNCaP-like’ tumors with EpMs and oncolytic viruses, as it would be possible to benefit from the anti-malignancy effects of both therapy agents.

Different lines of evidence support the notion that in cancer cells, epigenetic regulation of gene expression and the IFN response are functionally intertwined. For example, cell immortalization is associated with DNA-methylation-based silencing of genes connected to the interferon response [69, 70]. Concerning prostate cancer, down regulation of IFN-induced genes was shown to associate with tumor progression [61]. Furthermore, our analysis of patient derived samples [39], showed a greater methylation of ISGs relative to randomly selected genes, and a positive correlation between overall gene methylation and methylation of ISGs. Interestingly, the notion of anti-tumorigenic effects of IFN signaling and the DNA-methylation-based silencing of this response was also shown in recent studies using low-dose treatments of cancer cells with inhibitors of DNA-methylases [71, 72]. Such treatments were shown to induce the expression of endogenous retroviruses and exert anti-tumor effects via the induction of an IFN response.

Histone acetylation promotes gene expression by functioning as a molecular switch leading to transcription-permissive chromatin [73]. Histone deacetylase inhibitors (HDACs), such as TSA, have been employed to counter epigenetic silencing of gene expression, including in prostate cancer cells [18, 25]. However, the role of HDACs in the interferon response and the effects of HDAC inhibition on gene expression following IFN-stimuli, present a more complex scenario. For example, HDAC activity was proposed to be required for STAT1 signaling, recruitment of RNA polymerase to promoters of ISGs, ISG expression and anti-viral response [74–77]. In contrast, in other studies, HDAC inhibition with TSA resulted in constitutive de-repression of the IFNβ promoter and induction of antiviral activity in murine L929 cells [78]. Moreover, HDAC inhibition reactivated IFNƛ signaling in U87 glioma cells [79], further suggesting a negative role for HDACs in the antiviral response. The complexity of the effects of HDACs on IFN signaling was further exemplified by the fact that in U87 cells, IFNα-signaling was not affected by HDAC inhibition [79]. Thus, in different cellular contexts, HDAC inhibitors may promote, repress or leave unperturbed IFN-induced responses. Possible explanations for the discrepancy in effects exerted by TSA are: different roles performed by different HDACs [80], the acetylation of non-histone targets such as NF-κB or STAT1 [81, 82], or other cell-type specific differences. In the present study, we showed that TSA mediated de-repression of genes related to anti-viral response, resulting in IFN-mediated increase in expression of genes such as IRF7, and reduction in the susceptibility of LNCaP cells to viral infections.

In addition to cell-autonomous effects on tumor cells and their interaction with viruses, IFNs also govern interactions between the tumor and the immune system. This includes activation of receptors and co-receptors that mediate recognition of the cancer cell by the immune cells (e.g. MHC class I, [83]), as well as checkpoint inhibitors (e.g., PD-L1, [84, 85]). In addition, silencing of JAK1 in a variety of tumor cell types, or treatment of these cells with JAK inhibitors, induced increased secretion of IFNγ by co-cultured NK cells and enhanced susceptibility of the tumor cells to NK-mediated lysis [86]. Moreover, inhibition of expression of JAK1 or STAT1 abrogated the ability of IFNγ to upregulate PD-L1 in the tumor cell, and reduced the ability of these cells to hamper NK-mediated immunotherapy [87]. Notably, LNCaP cells, which are defective in IFN signaling, present low expression of MHC class I, that exposes them to NK-mediated killing [88–90]. The potentiation of NK-mediated anti-tumor effects by oncolytic viruses has begun to be tested [91]. Along these lines, a recent study employing NDV as oncolytic virus showed that combination therapy with NDV and anti–CTLA-4 induces infiltration of distant (and uninfected tumors) with activated lymphocytes [92]. Together, these studies suggest the possibility of exploiting the synergy between therapeutic effects of oncolytic virotherapy and immunotherapy. We propose that the IFN-refractory subset of tumors (i.e., similar to LNCaP cells) may be particularly prone to such benefits.

## MATERIALS AND METHODS

### Cell culture and viruses

Lymph node carcinoma of the prostate (LNCaP) cells and DU145 cells were gifts from Prof. Pinkas-Kramarski (Tel Aviv University). The identity of cells was confirmed by STR analysis at the biomedical core facility at the genomic center (Technion, Israel). non-transformed human telomerase reverse transcriptase (hTERT)-immortalized prostate cancer cells (EP, [59] a gift from Dr. Raanan Berger, Tel HaShomer Hospital, Israel). Baby Hamster Kidney cells (BHK-21, ATCC) were employed for plaque assays. Cells were grown in either modified Eagle's medium (MEM, BHK-21), Prostate Epithelial Growth Medium (Lonza, EP), Roswell Park Memorial Institute medium (RPMI 1640, LNCaP), or Dulbecco's modified Eagle's medium (DMEM, DU145) supplemented with 10% Fetal Calf Serum, 5 mM Glutamine and Penicillin-Streptomycin (all from Beit Haemek Biological Industries, Israel) at 37°C and 5% CO_2_. Construction and propagation of hMPV-GFP clone were described previously [31]. hMPV-GFP infections were carried in infection media: RPMI (LNCaP) or DMEM (DU145) supplemented with 3% Fetal Calf Serum, 5 mM Glutamine and Penicillin-Streptomycin and 0.25 mg/ml Trypsin. To determine titer of hMPV-GFP, LNCaP cells were grown to 70% confluency in 12 well plate; incubated with serial dilutions of the virus in infection media for 2 hours, prior to incubation of cells in medium supplemented with 10% FCS without trypsin for 24 hours. Cells were subsequently trypsinized, fixed in 2% paraformaldehyde and analyzed by fluorescence-activated cell sorting (FACS) to quantify the total number of GFP-positive cells. EHDV-TAU was generated by serial passaging of EHDV2-Ibaraki isolate [34] on LNCaP cells for 16 times. Each of the 16 repetitions included: (i) infection of LNCaP cells; (ii) lysis of infected cells by sonication; (iii) plaque assay of the viral progeny on naive LNCaP cells; (iv) plaque-purification of the virus from largest plaques, for re-infection of LNCaP cells.

### Plaque assay

EHDV-TAU or EHDV2-Ibaraki were collected from infected LNCaP cell cultures (medium + cells). Virus was released from attached and detached cells by sonication. Serial dilutions were used to infect reporter cultures (5*10^5^ BHK cells/well; seeded in 12 well plates). One well was left uninfected as control. Plates were incubated with virus at 37°C for 1 h, after which cells were washed and overlayed with 0.3% tragacanth (Sigma-Aldrich, cat. # G1128, St. Louis, Mo, USA) in MEM. After 4 days, cultures were fixed with crystal violet (Sigma-Aldrich, cat. # C0775, St. Louis, Mo, USA)/formaldehyde. Virus titer (PFU/ml) was calculated according to number of plaques and dilution factor.

### Antibodies

Anti-NS3 antibodies were described in [34]. Rabbit anti-phospho-Tyr701-STAT1, rabbit anti-STAT1, rabbit anti-phospho-NF-κB p65 (Ser 536) and rabbit anti-NF-κB p65, diluted 1:1000 for western blot and 1:200 for immunofluorescence, were from Cell Signaling (Beverly, MA, USA). Mouse anti-Actin, diluted 1:10000, was from MP Biomedicals (Santa Ana, CA, USA, cat. #69100). Mouse anti-tubulin-α, diluted 1:1000, was from Biolegend (San Diego, CA, USA, cat. #625901). Alexa-488 and Alexa-555 conjugated secondary antibodies, diluted 1:200, were from ThermoFisher (cat. #A27039 and A28175). HRP-conjugated secondary antibodies, diluted 1:15,000, were from Jackson ImmunoResearch Laboratories (West Grove, PA, USA, cat. #115035003).

### Drugs and reagents

Reagents were employed at the following final concentrations: RG108- 200 μM; 5-Aza-2′-deoxycytidine (5AC) - 20 μM; Trichostatin A (TSA) - 0.1 μM, all purchased from Sigma-Aldrich (Sigma-Aldrich, St. Louis, Mo, USA, cat. #R8279, A3656 and T8552). Human interferon (IFNαB2), 200 U/ml (PBL-assay science, Piscataway, NJ, USA, cat. #111051); DAPI (4′, 6-Diamidino-2-Phenylindole, Dihydrochloride) 1 μg/ml was from Sigma-Aldrich (St. Louis, Mo, USA, cat. # D9542). Human TNF-α-10ng/ml (PeproTech NJ, USA, cat. # 30001A).

### FACS analysis

LNCaP cells, infected and treated with the indicated drugs, were fixed with 2% paraformaldehyde and analyzed by FACS for GFP fluorescence, using a FACSort apparatus (Becton Dickinson). Each independent experiment included also (i) uninfected LNCaP cells for background autofluorescence, (ii) hMPV-infected, non-treated LNCaP cells. FACS analysis was done with FlowJo software.

### Immunoblotting

Cell pellets were lysed in ice-cold RIPA buffer (150 mM NaCl, 1% NP-40, 0.5% deoxycholate, 0.1% sodium dodecylsulfate, 50 mM Tris-HCl pH 8.0) supplemented with protease inhibitor (Complete Protease Inhibitor Cocktail; Roche, cat. #11-697-498) and phosphatase inhibitor (Phosphatase Inhibitor Cocktail 2+3 (Sigma-Aldrich cat. #p5726, p0044) for 30 minutes on ice. Lysates were cleared by centrifugation (15 minutes, 16,000 x g, 4°C). For each lysate, protein concentration was determined using the Pierce BCA Protein Assay Kit (ThermoFisher, cat. #23225). 10-50 μg of protein (depending on experiment) were separated by sodium dodecyl sulfate polyacrylamide gel electrophoresis (SDS-PAGE) through 10% polyacrylamide gels and transferred to an Immobilon-P membrane (Millipore) according to the manufacturer's instructions. Membranes were blocked for 1 hour in TBST buffer (0.05 M Tris-HCl pH 7.5, 0.15 M NaCl, and 0.1% Tween 20) containing 5% milk, and blotted with primary antibodies overnight at 4°C. Secondary antibody linked to horseradish peroxidase was then added for 1 hour. Immunoreactive bands were detected with the Enhanced Chemiluminescence Substrate (Western Lightning Plus-ECL; PerkinElmer, cat. #NEL105001EA).

### qRT-PCR

Total RNA was extracted from cells using EZ-RNA kit (Biological Industries, Israel, cat. #20-400-100) according to the manufacturer's instructions. First-strand cDNA synthesis was performed using the iScript cDNA Synthesis Kit (Bio Rad cat. #1708890) according to the manufacturer's instructions, with additional no-RNA control. Real-Time PCR analyses of STAT1, IRF7, MX1 DUSP5, PKR and JAK1 mRNA levels, relative to GAPDH mRNA levels were performed in triplicates, using Fast SYBR-green master mix (Applied Biosystems, cat.#4385612) with StepOnePlus Real-Time PCR System (Applied Biosystems, cat. #4376600). Primers were designed to span exon-exon junctions and to produce 80-140bp amplicons. Primers employed are listed in Supplementary Table 1. Gene expression values were calculated based on the comparative threshold cycle method [93].

### Genomic DNA sequencing

Genomic DNA of LNCaP or DU145 was extracted with GenElute Mammalian Genomic DNA Miniprep Kit (Sigma, cat. #G1N70). Exons 5 and 9 of JAK1 (where the first exon of JAK1 sequence - NM_002227 - is counted as 1) were amplified from purified genomic DNA preparations, using the cognate primers (Supplementary Table 2) and Phusion High-Fidelity DNA Polymerase (NEB, cat. #M0530S). PCR products were separated by electrophoresis in agarose gels and purified using gel extraction kit (Qiagen, cat. #28704). Sequencing of the PCR products was done with ABI 3100 Genetic Analyzer machine.

### Cloning

LNCaP cells were treated with 5AC and TSA, as described above, and total RNA was extracted. This treatment was required to induce JAK1 mRNA to levels sufficient for cloning. cDNA was prepared as above and amplified with Phusion Polymerase (NEB), with primers flanking JAK1 exon 5 and exon 9 (Supplementary Table 2). PCR products were gel-purified using gel extraction kit (Qiagen), and cloned into pJET1.2/blunt vector (CloneJET PCR Cloning Kitl; ThermoFisher, cat. #K1231). Plasmids were purified with GenElute Plasmid Miniprep Kit (Sigma, cat. #PLN10), and inserts were sequenced with the corresponding pJET primers with an ABI 3100 Genetic Analyzer machine.

### Microscopy

Images were acquired with a spinning disk confocal microscope (CSU-22 Confocal Head, Yokogawa; Axiovert 200M, Carl Zeiss MicroImaging) under control of SlideBook (Intelligent Imaging Innovations), with 63x oil immersion objective (Plan Apochromat, NA 1.4), Evolve camera (Photometrics) and laser illumination; or 10x air objective (Plan Apochromat, NA 0.25), EZ camera (Photometrics) and illumination with fluorescence lamp.

### Live microscopy

4*10^5^ LNCaP cells were plated on a 35mm tissue-culture plate. 12-16 hours after plating, the cells were treated, or not, with EpMs (with or without IFNα, which was added 4 hours prior to infection) for 24 hours. hMPV-GFP infection was done in a final volume of 2ml of infection media, supplemented with 50mM HEPES. The cells were placed in a 37°C chamber and bright-field and fluorescence images were taken in intervals of 10 min during 24 hours after infection. For analysis of GFP signal intensity, images were segmented according to specific GFP-signal intensity (same value for all conditions), and the values obtained were normalized to the number of cells in each timelapse. Analysis was done using the SlideBook (Intelligent Imaging Innovations) software.

### Immunofluorescence

DU145 or LNCaP were seeded (5*10^4^ cells/well) onto glass coverslips placed in a 24-well plate, and were infected, or not, with EHDV-TAU, in the presence or absence of IFNα. At 44 hours post infection (hpi) or 2 h (for IFNα treatment), cells were washed twice with cold PBS (4°C), fixed (4% paraformaldehyde, 20 min), blocked and permeabilized [30 min, PBS/BSA/T (PBS, 1% BSA, 0.1% triton)], and stained with polyclonal anti-NS3 (1:300 dilution in PBS/BSA/T). Alexa-488 or 555-conjugated goat-anti-rabbit antibodies (1:200 dilution in PBS/BSA/T) were used as secondary antibodies. To detect nuclear phosphorylated STAT1, cells were fixed (4 % paraformaldehyde, 20 min) and permeabilized with ice-cold methanol (10min −20°). Cells were stained with Rabbit-anti phospho-Tyr701-STAT1 (1:100 dilution in PBS/BSA/T) for overnight at 4°C. Mounting was with Fluorescence Mounting Medium (Golden Bridge, Mukilteo, WA, USA, cat. #E1818).

### Trypan blue exclusion assay

2×10^5^ LNCaP cells/well in a 12-well plate were infected, or not, under the indicated experimental conditions. For each well, detached and attached cells were collected together and mixed with 0.5% trypan blue at a 1:1 ratio. Cells were classified by trypan blue exclusion by light microscopy. Trypan blue (0.5 %) was from Beit Haemek Biological Industries, Israel (cat. #03-102-1B).

### Generation of a list of randomized 500 human genes

19001 protein coding genes were downloaded from HGNC site (HUGO gene nomenclature committee, [94]) to an Excel sheet. The Excel RAND function was used to generate a randomize number for each of the genes, which were then sorted from the smallest to the largest value. The first 500 hundred genes were selected for farther analyses.

### Luciferase assay

DU145 and LNCaP cells were co-transfected with pISRE-Luc (Clontech, PT3372-5W) and renilla luciferase plasmid (pRL-TK; Promega, E2241) as a control. pISRE-Luc contains the firefly luciferase gene under the control of five copies of the ISRE-binding sequence, located upstream of the TATA-like promoter of the herpes simplex virus thymidine kinase. Transfections were carried out with PolyJet *In Vitro* DNA Tranfection Reagent (SignaGen Laboratories, SL100688). Luciferase activity was detected with the Dual-Luciferase Reporter Assay System (Promega, E1910).

### Statistical analysis

Data are expressed as means ± standard deviation (SD). Significant differences in mean values were assessed by 1-tailed Student's t-test. A value of p ≤ 0.05 was considered significant. All experiments were repeated at least three times.

## SUPPLEMENTARY INFORMATION























## References

[1] Siegel R, Naishadham D, Jemal A (2013). Cancer statistics, 2013. CA Cancer J Clin.

[2] Boyd LK, Mao X, Lu YJ (2012). The complexity of prostate cancer: genomic alterations and heterogeneity. Nature reviews Urology.

[3] White NM, Feng FY, Maher CA (2013). Recurrent rearrangements in prostate cancer: causes and therapeutic potential. Current drug targets.

[4] Tomlins SA, Laxman B, Dhanasekaran SM, Helgeson BE, Cao X, Morris DS, Menon A, Jing X, Cao Q, Han B, Yu J, Wang L, Montie JE, Rubin MA, Pienta KJ, Roulston D (2007). Distinct classes of chromosomal rearrangements create oncogenic ETS gene fusions in prostate cancer. Nature.

[5] Varambally S, Dhanasekaran SM, Zhou M, Barrette TR, Kumar-Sinha C, Sanda MG, Ghosh D, Pienta KJ, Sewalt RG, Otte AP, Rubin MA, Chinnaiyan AM (2002). The polycomb group protein EZH2 is involved in progression of prostate cancer. Nature.

[6] Yang YA, Yu J (2013). EZH2, an epigenetic driver of prostate cancer. Protein & cell.

[7] da Silva HB, Amaral EP, Nolasco EL, de Victo NC, Atique R, Jank CC, Anschau V, Zerbini LF, Correa RG (2013). Dissecting Major Signaling Pathways throughout the Development of Prostate Cancer. Prostate cancer.

[8] O'Shea JJ, Schwartz DM, Villarino AV, Gadina M, McInnes IB, Laurence A (2015). The JAK-STAT pathway: impact on human disease and therapeutic intervention. Annual review of medicine.

[9] Schneider WM, Chevillotte MD, Rice CM (2014). Interferon-stimulated genes: a complex web of host defenses. Annual review of immunology.

[10] Schoggins JW (2014). Interferon-stimulated genes: roles in viral pathogenesis. Current opinion in virology.

[11] Sica G, Fabbroni L, Castagnetta L, Cacciatore M, Pavone-Macaluso M (1989). Antiproliferative effect of interferons on human prostate carcinoma cell lines. Urological research.

[12] van Haelst-Pisani CM, Richardson RL, Su J, Buckner JC, Hahn RG, Frytak S, Kvols LK, Burch PA (1992). A phase II study of recombinant human alpha-interferon in advanced hormone-refractory prostate cancer. Cancer.

[13] Tsaur I, Hudak L, Makarevic J, Juengel E, Mani J, Borgmann H, Gust KM, Schilling D, Bartsch G, Nelson K, Haferkamp A, Blaheta RA (2015). Intensified antineoplastic effect by combining an HDAC-inhibitor, an mTOR-inhibitor, low dosed interferon alpha in prostate cancer cells. Journal of cellular and molecular medicine.

[14] Tan H, Zeng C, Xie J, Alghamdi NJ, Song Y, Zhang H, Zhou A, Jin D (2015). Effects of interferons and double-stranded RNA on human prostate cancer cell apoptosis. Oncotarget.

[15] Daliani DD, Eisenberg PD, Weems J, Lord R, Fueger R, Logothetis CJ (1995). The results of a phase II randomized trial comparing 5-fluorouracil and 5-fluorouracil plus alpha-interferon: observations on the design of clinical trials for androgen-independent prostate cancer. The Journal of urology.

[16] Rossi MR, Hawthorn L, Platt J, Burkhardt T, Cowell JK, Ionov Y (2005). Identification of inactivating mutations in the JAK1, SYNJ2, and CLPTM1 genes in prostate cancer cells using inhibition of nonsense-mediated decay and microarray analysis. Cancer genetics and cytogenetics.

[17] Dunn GP, Sheehan KC, Old LJ, Schreiber RD (2005). IFN unresponsiveness in LNCaP cells due to the lack of JAK1 gene expression. Cancer research.

[18] Ibragimova I, Ibanez de Caceres I, Hoffman AM, Potapova A, Dulaimi E, Al-Saleem T, Hudes GR, Ochs MF, Cairns P (2010). Global reactivation of epigenetically silenced genes in prostate cancer. Cancer prevention research.

[19] Jerónimo C, Bastian PJ, Bjartell A, Carbone GM, Catto JWF, Clark SJ, Henrique R, Nelson WG, Shariat SF (2011). Epigenetics in Prostate Cancer: Biologic and Clinical Relevance. European urology.

[20] Valdes-Mora F, Clark SJ (2015). Prostate cancer epigenetic biomarkers: next-generation technologies. Oncogene.

[21] Ilkow CS, Swift SL, Bell JC, Diallo JS (2014). From scourge to cure: tumour-selective viral pathogenesis as a new strategy against cancer. PLoS pathogens.

[22] Horoszewicz JS, Leong SS, Chu TM, Wajsman ZL, Friedman M, Papsidero L, Kim U, Chai LS, Kakati S, Arya SK, Sandberg AA (1980). The LNCaP cell line--a new model for studies on human prostatic carcinoma. Progress in clinical and biological research.

[23] Cunningham D, You Z (2015). *In vitro* and *in vivo* model systems used in prostate cancer research. Journal of biological methods.

[24] Tai S, Sun Y, Squires JM, Zhang H, Oh WK, Liang CZ, Huang J (2011). PC3 is a cell line characteristic of prostatic small cell carcinoma. The Prostate.

[25] Lodygin D, Epanchintsev A, Menssen A, Diebold J, Hermeking H (2005). Functional epigenomics identifies genes frequently silenced in prostate cancer. Cancer research.

[26] Kim JH, Dhanasekaran SM, Prensner JR, Cao X, Robinson D, Kalyana-Sundaram S, Huang C, Shankar S, Jing X, Iyer M, Hu M, Sam L, Grasso C, Maher CA, Palanisamy N, Mehra R (2011). Deep sequencing reveals distinct patterns of DNA methylation in prostate cancer. Genome research.

[27] Carey BL, Ahmed M, Puckett S, Lyles DS (2008). Early steps of the virus replication cycle are inhibited in prostate cancer cells resistant to oncolytic vesicular stomatitis virus. Journal of virology.

[28] Thirukkumaran CM, Nodwell MJ, Hirasawa K, Shi ZQ, Diaz R, Luider J, Johnston RN, Forsyth PA, Magliocco AM, Lee P, Nishikawa S, Donnelly B, Coffey M, Trpkov K, Fonseca K, Spurrell J (2010). Oncolytic viral therapy for prostate cancer: efficacy of reovirus as a biological therapeutic. Cancer research.

[29] Liu C, Hasegawa K, Russell SJ, Sadelain M, Peng KW (2009). Prostate-specific membrane antigen retargeted measles virotherapy for the treatment of prostate cancer. The Prostate.

[30] Msaouel P, Iankov ID, Allen C, Morris JC, von Messling V, Cattaneo R, Koutsilieris M, Russell SJ, Galanis E (2009). Engineered measles virus as a novel oncolytic therapy against prostate cancer. The Prostate.

[31] Sabo Y, Ehrlich M, Bacharach E (2011). The conserved YAGL motif in human metapneumovirus is required for higher-order cellular assemblies of the matrix protein and for virion production. Journal of virology.

[32] Banos-Lara Mdel R, Harvey L, Mendoza A, Simms D, Chouljenko VN, Wakamatsu N, Kousoulas KG, Guerrero-Plata A (2015). Impact and regulation of lambda interferon response in human metapneumovirus infection. Journal of virology.

[33] Savini G, Afonso A, Mellor P, Aradaib I, Yadin H, Sanaa M, Wilson W, Monaco F, Domingo M (2011). Epizootic heamorragic disease. Research in veterinary science.

[34] Shai B, Schmukler E, Yaniv R, Ziv N, Horn G, Bumbarov V, Yadin H, Smorodinsky NI, Bacharach E, Pinkas-Kramarski R, Ehrlich M (2013). Epizootic hemorrhagic disease virus induces and benefits from cell stress, autophagy, and apoptosis. Journal of virology.

[35] Vitour D, Doceul V, Ruscanu S, Chauveau E, Schwartz-Cornil I, Zientara S (2014). Induction and control of the type I interferon pathway by Bluetongue virus. Virus research.

[36] Chauveau E, Doceul V, Lara E, Adam M, Breard E, Sailleau C, Viarouge C, Desprat A, Meyer G, Schwartz-Cornil I, Ruscanu S, Charley B, Zientara S, Vitour D (2012). Sensing and control of bluetongue virus infection in epithelial cells via RIG-I and MDA5 helicases. Journal of virology.

[37] Gao J, Aksoy BA, Dogrusoz U, Dresdner G, Gross B, Sumer SO, Sun Y, Jacobsen A, Sinha R, Larsson E, Cerami E, Sander C, Schultz N (2013). Integrative analysis of complex cancer genomics and clinical profiles using the cBioPortal. Science signaling.

[38] Cerami E, Gao J, Dogrusoz U, Gross BE, Sumer SO, Aksoy BA, Jacobsen A, Byrne CJ, Heuer ML, Larsson E, Antipin Y, Reva B, Goldberg AP, Sander C, Schultz N (2012). The cBio cancer genomics portal: an open platform for exploring multidimensional cancer genomics data. Cancer discovery.

[39] Cancer Genome Atlas Research N (2015). The Molecular Taxonomy of Primary Prostate Cancer. Cell.

[40] Pan Y, Kytola S, Farnebo F, Wang N, Lui WO, Nupponen N, Isola J, Visakorpi T, Bergerheim US, Larsson C (1999). Characterization of chromosomal abnormalities in prostate cancer cell lines by spectral karyotyping. Cytogenetics and cell genetics.

[41] van Bokhoven A, Caires A, Maria MD, Schulte AP, Lucia MS, Nordeen SK, Miller GJ, Varella-Garcia M (2003). Spectral karyotype (SKY) analysis of human prostate carcinoma cell lines. The Prostate.

[42] Mishra DK, Chen Z, Wu Y, Sarkissyan M, Koeffler HP, Vadgama JV (2010). Global methylation pattern of genes in androgen-sensitive and androgen-independent prostate cancer cells. Molecular cancer therapeutics.

[43] Schoggins JW, Wilson SJ, Panis M, Murphy MY, Jones CT, Bieniasz P, Rice CM (2011). A diverse range of gene products are effectors of the type I interferon antiviral response. Nature.

[44] Tomlins SA, Rhodes DR, Perner S, Dhanasekaran SM, Mehra R, Sun XW, Varambally S, Cao X, Tchinda J, Kuefer R, Lee C, Montie JE, Shah RB, Pienta KJ, Rubin MA, Chinnaiyan AM (2005). Recurrent fusion of TMPRSS2 and ETS transcription factor genes in prostate cancer. Science.

[45] Alimirah F, Chen J, Davis FJ, Choubey D (2007). IFI16 in human prostate cancer. Molecular cancer research.

[46] Brueckner B, Garcia Boy R, Siedlecki P, Musch T, Kliem HC, Zielenkiewicz P, Suhai S, Wiessler M, Lyko F (2005). Epigenetic reactivation of tumor suppressor genes by a novel small-molecule inhibitor of human DNA methyltransferases. Cancer research.

[47] Yang CH, Murti A, Valentine WJ, Du Z, Pfeffer LM (2005). Interferon alpha activates NF-kappaB in JAK1-deficient cells through a TYK2-dependent pathway. The Journal of biological chemistry.

[48] Platanias LC (2005). Mechanisms of type-I- and type-II-interferon-mediated signalling. Nature reviews Immunology.

[49] Cai C, Chen JY, Han ZD, He HC, Chen JH, Chen YR, Yang SB, Wu YD, Zeng YR, Zou J, Liang YX, Dai QS, Jiang FN, Zhong WD (2015). Down-regulation of dual-specificity phosphatase 5 predicts poor prognosis of patients with prostate cancer. International journal of clinical and experimental medicine.

[50] Mushinski JF, Nguyen P, Stevens LM, Khanna C, Lee S, Chung EJ, Lee MJ, Kim YS, Linehan WM, Horisberger MA, Trepel JB (2009). Inhibition of tumor cell motility by the interferon-inducible GTPase MxA. The Journal of biological chemistry.

[51] Roberts MS, Lorence RM, Groene WS, Bamat MK (2006). Naturally oncolytic viruses. Current opinion in molecular therapeutics.

[52] Shmulevitz M, Marcato P, Lee PW (2005). Unshackling the links between reovirus oncolysis, Ras signaling, translational control and cancer. Oncogene.

[53] Donnelly OG, Errington-Mais F, Prestwich R, Harrington K, Pandha H, Vile R, Melcher AA (2012). Recent clinical experience with oncolytic viruses. Current pharmaceutical biotechnology.

[54] Maitra R, Ghalib MH, Goel S (2012). Reovirus: a targeted therapeutic--progress and potential. Molecular cancer research.

[55] Freeman AI, Zakay-Rones Z, Gomori JM, Linetsky E, Rasooly L, Greenbaum E, Rozenman-Yair S, Panet A, Libson E, Irving CS, Galun E, Siegal T (2006). Phase I/II trial of intravenous NDV-HUJ oncolytic virus in recurrent glioblastoma multiforme. Molecular therapy.

[56] Stojdl DF, Lichty B, Knowles S, Marius R, Atkins H, Sonenberg N, Bell JC (2000). Exploiting tumor-specific defects in the interferon pathway with a previously unknown oncolytic virus. Nature medicine.

[57] Stojdl DF, Lichty BD, tenOever BR, Paterson JM, Power AT, Knowles S, Marius R, Reynard J, Poliquin L, Atkins H, Brown EG, Durbin RK, Durbin JE, Hiscott J, Bell JC (2003). VSV strains with defects in their ability to shutdown innate immunity are potent systemic anti-cancer agents. Cancer cell.

[58] Chan WM, Rahman MM, McFadden G (2013). Oncolytic myxoma virus: the path to clinic. Vaccine.

[59] Leshem O, Madar S, Kogan-Sakin I, Kamer I, Goldstein I, Brosh R, Cohen Y, Jacob-Hirsch J, Ehrlich M, Ben-Sasson S, Goldfinger N, Loewenthal R, Gazit E, Rotter V, Berger R (2011). TMPRSS2/ERG promotes epithelial to mesenchymal transition through the ZEB1/ZEB2 axis in a prostate cancer model. PloS one.

[60] Degterev A, Huang Z, Boyce M, Li Y, Jagtap P, Mizushima N, Cuny GD, Mitchison TJ, Moskowitz MA, Yuan J (2005). Chemical inhibitor of nonapoptotic cell death with therapeutic potential for ischemic brain injury. Nature chemical biology.

[61] Shou J, Soriano R, Hayward SW, Cunha GR, Williams PM, Gao WQ (2002). Expression profiling of a human cell line model of prostatic cancer reveals a direct involvement of interferon signaling in prostate tumor progression. Proceedings of the National Academy of Sciences of the United States of America.

[62] Hastie C (2008). Interferon gamma, a possible therapeutic approach for late-stage prostate cancer?. Anticancer research.

[63] Hudak L, Tezeeh P, Wedel S, Makarevic J, Juengel E, Tsaur I, Bartsch G, Wiesner C, Haferkamp A, Blaheta RA (2012). Low dosed interferon alpha augments the anti-tumor potential of histone deacetylase inhibition on prostate cancer cell growth and invasion. The Prostate.

[64] Persano L, Moserle L, Esposito G, Bronte V, Barbieri V, Iafrate M, Gardiman MP, Larghero P, Pfeffer U, Naschberger E, Sturzl M, Indraccolo S, Amadori A (2009). Interferon-alpha counteracts the angiogenic switch and reduces tumor cell proliferation in a spontaneous model of prostatic cancer. Carcinogenesis.

[65] Taylor CW, Modiano MR, Woodson ME, Marcus SG, Alberts DS, Hersh EM (1992). A phase I trial of fluorouracil, leucovorin, and recombinant interferon alpha-2b in patients with advanced malignancy. Seminars in oncology.

[66] Strefford JC, Lillington DM, Young BD, Oliver RT (2001). The use of multicolor fluorescence technologies in the characterization of prostate carcinoma cell lines: a comparison of multiplex fluorescence in situ hybridization and spectral karyotyping data. Cancer genetics and cytogenetics.

[67] Sehgal R, Sheahan K, O'Connell PR, Hanly AM, Martin ST, Winter DC (2014). Lynch syndrome: an updated review. Genes.

[68] Eilers A, Kanda K, Klose B, Krolewski J, Decker T (1996). Constitutive STAT1 tyrosine phosphorylation in U937 monocytes overexpressing the TYK2 protein tyrosine kinase does not induce gene transcription. Cell growth & differentiation.

[69] Fridman AL, Tang L, Kulaeva OI, Ye B, Li Q, Nahhas F, Roberts PC, Land SJ, Abrams J, Tainsky MA (2006). Expression profiling identifies three pathways altered in cellular immortalization: interferon, cell cycle, and cytoskeleton. The journals of gerontology Series A, Biological sciences and medical sciences.

[70] Kulaeva OI, Draghici S, Tang L, Kraniak JM, Land SJ, Tainsky MA (2003). Epigenetic silencing of multiple interferon pathway genes after cellular immortalization. Oncogene.

[71] Roulois D, Loo Yau H, Singhania R, Wang Y, Danesh A, Shen SY, Han H, Liang G, Jones PA, Pugh TJ, O'Brien C, De Carvalho DD (2015). DNA-Demethylating Agents Target Colorectal Cancer Cells by Inducing Viral Mimicry by Endogenous Transcripts. Cell.

[72] Chiappinelli KB, Strissel PL, Desrichard A, Li H, Henke C, Akman B, Hein A, Rote NS, Cope LM, Snyder A, Makarov V, Buhu S, Slamon DJ, Wolchok JD, Pardoll DM, Beckmann MW (2015). Inhibiting DNA Methylation Causes an Interferon Response in Cancer via dsRNA Including Endogenous Retroviruses. Cell.

[73] Eberharter A, Becker PB (2002). Histone acetylation: a switch between repressive and permissive chromatin. Second in review series on chromatin dynamics. EMBO reports.

[74] Nusinzon I, Horvath CM (2003). Interferon-stimulated transcription and innate antiviral immunity require deacetylase activity and histone deacetylase 1. Proceedings of the National Academy of Sciences of the United States of America.

[75] Klampfer L, Huang J, Swaby LA, Augenlicht L (2004). Requirement of histone deacetylase activity for signaling by STAT1. The Journal of biological chemistry.

[76] Chang HM, Paulson M, Holko M, Rice CM, Williams BR, Marie I, Levy DE (2004). Induction of interferon-stimulated gene expression and antiviral responses require protein deacetylase activity. Proceedings of the National Academy of Sciences of the United States of America.

[77] Sakamoto S, Potla R, Larner AC (2004). Histone deacetylase activity is required to recruit RNA polymerase II to the promoters of selected interferon-stimulated early response genes. The Journal of biological chemistry.

[78] Shestakova E, Bandu MT, Doly J, Bonnefoy E (2001). Inhibition of histone deacetylation induces constitutive derepression of the beta interferon promoter and confers antiviral activity. Journal of virology.

[79] Ding S, Khoury-Hanold W, Iwasaki A, Robek MD (2014). Epigenetic reprogramming of the type III interferon response potentiates antiviral activity and suppresses tumor growth. PLoS biology.

[80] Nusinzon I, Horvath CM (2006). Positive and negative regulation of the innate antiviral response and beta interferon gene expression by deacetylation. Molecular and cellular biology.

[81] Kramer OH, Baus D, Knauer SK, Stein S, Jager E, Stauber RH, Grez M, Pfitzner E, Heinzel T (2006). Acetylation of Stat1 modulates NF-kappaB activity. Genes & development.

[82] Zhang Q, Yang F, Li X, Wang LW, Chu XG, Zhang H, Gong ZJ (2015). Trichostatin A Protects Against Experimental Acute-on-Chronic Liver Failure in Rats Through Regulating the Acetylation of Nuclear Factor-kappaB. Inflammation.

[83] Wan S, Pestka S, Jubin RG, Lyu YL, Tsai YC, Liu LF (2012). Chemotherapeutics and radiation stimulate MHC class I expression through elevated interferon-beta signaling in breast cancer cells. PloS one.

[84] Eppihimer MJ, Gunn J, Freeman GJ, Greenfield EA, Chernova T, Erickson J, Leonard JP (2002). Expression and regulation of the PD-L1 immunoinhibitory molecule on microvascular endothelial cells. Microcirculation (New York, NY: 1994).

[85] Schreiner B, Mitsdoerffer M, Kieseier BC, Chen L, Hartung HP, Weller M, Wiendl H (2004). Interferon-beta enhances monocyte and dendritic cell expression of B7-H1 (PD-L1), a strong inhibitor of autologous T-cell activation: relevance for the immune modulatory effect in multiple sclerosis. Journal of neuroimmunology.

[86] Bellucci R, Nguyen HN, Martin A, Heinrichs S, Schinzel AC, Hahn WC, Ritz J (2012). Tyrosine kinase pathways modulate tumor susceptibility to natural killer cells. The Journal of clinical investigation.

[87] Bellucci R, Martin A, Bommarito D, Wang K, Hansen SH, Freeman GJ, Ritz J (2015). Interferon-gamma-induced activation of JAK1 and JAK2 suppresses tumor cell susceptibility to NK cells through upregulation of PD-L1 expression. Oncoimmunology.

[88] Sanda MG, Restifo NP, Walsh JC, Kawakami Y, Nelson WG, Pardoll DM, Simons JW (1995). Molecular characterization of defective antigen processing in human prostate cancer. Journal of the National Cancer Institute.

[89] Long EO, Kim HS, Liu D, Peterson ME, Rajagopalan S (2013). Controlling natural killer cell responses: integration of signals for activation and inhibition. Annual review of immunology.

[90] Ogbomo H, Michaelis M, Geiler J, van Rikxoort M, Muster T, Egorov A, Doerr HW, Cinatl J (2010). Tumor cells infected with oncolytic influenza A virus prime natural killer cells for lysis of resistant tumor cells. Medical microbiology and immunology.

[91] Rintoul JL, Lemay CG, Tai LH, Stanford MM, Falls TJ, de Souza CT, Bridle BW, Daneshmand M, Ohashi PS, Wan Y, Lichty BD, Mercer AA, Auer RC, Atkins HL, Bell JC (2012). ORFV: a novel oncolytic and immune stimulating parapoxvirus therapeutic. Molecular therapy.

[92] Zamarin D, Holmgaard RB, Subudhi SK, Park JS, Mansour M, Palese P, Merghoub T, Wolchok JD, Allison JP (2014). Localized oncolytic virotherapy overcomes systemic tumor resistance to immune checkpoint blockade immunotherapy. Science translational medicine.

[93] Livak KJ, Schmittgen TD (2001). Analysis of relative gene expression data using real-time quantitative PCR and the 2(-Delta Delta C(T)) Method. Methods.

[94] Gray KA, Yates B, Seal RL, Wright MW, Bruford EA (2015). Genenames.org: the HGNC resources in 2015. Nucleic acids research.

