# Supplementary material for: Combined genetic and epigenetic interferences with interferon signaling expose prostate cancer cells to viral infection
Source: Oncotarget. 2016 Jun 28;7(32):52115–34. doi: 10.18632/oncotarget.10313 (PMC5239539; doi:10.18632/oncotarget.10313)
Supplement: Supplementary file 1 [file oncotarget-07-52115-s001.pdf]

Combined genetic and epigenetic interferences with interferon signaling expose prostate cancer cells to viral infection

SUPPLEMENTARY INFORMATION

Supplementary Movies S1 to S8: Live cell microscopy (fluorescence microscopy superimposed on phase contrast microscopy) of hMPV-GFP infection of LNCaP cells under different conditions of EpM and/or IFNα treatments. All timelapse sequences were acquired under identical conditions (same exposures in bright-field and GFP channels; frequency 1 frame per 10 min). Movies are numbered: S1, untreated infected; S2, IFNα treated; S3, RG108; S4, RG108 + IFNα; S5, 5AC; S6, 5AC + IFNα; S7, TSA; S8, TSA + IFNα.

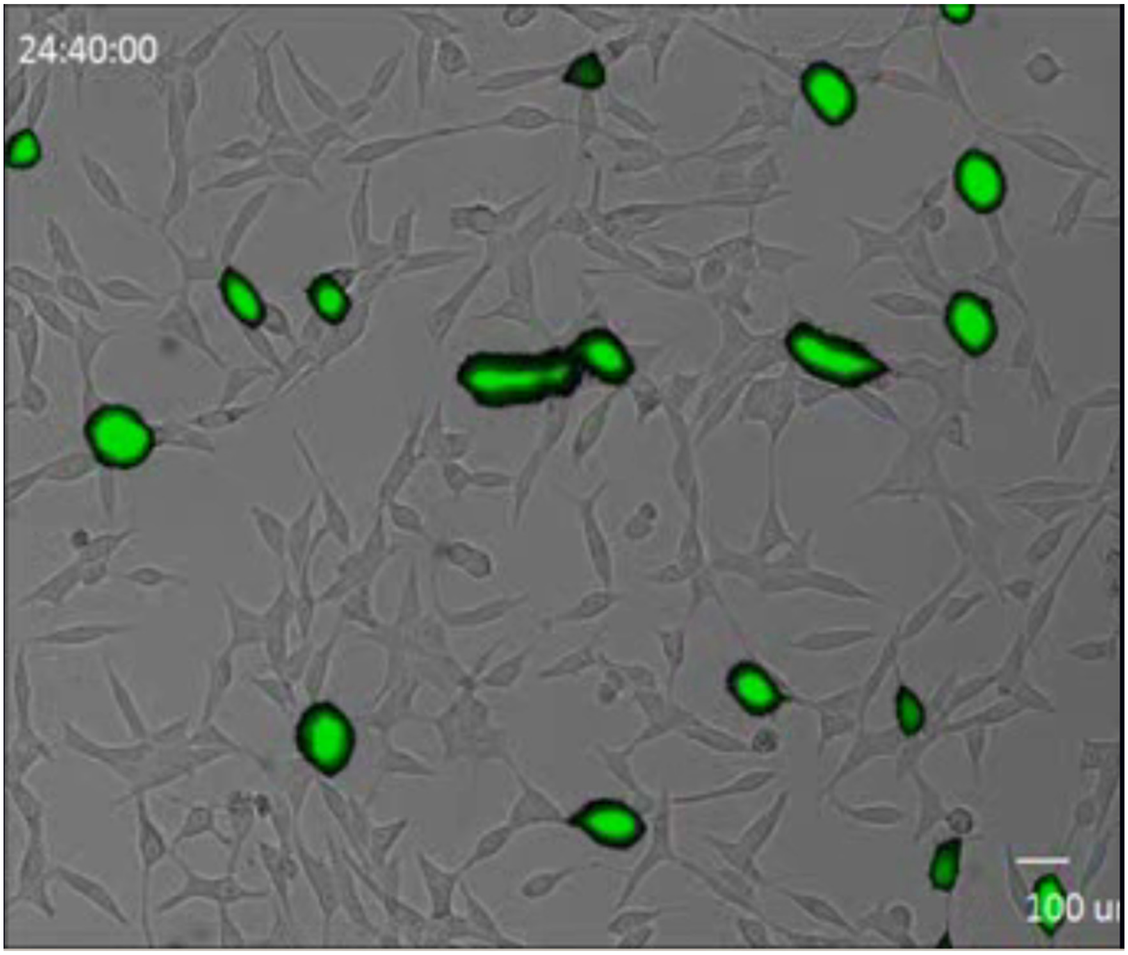

See Supplementary File 1

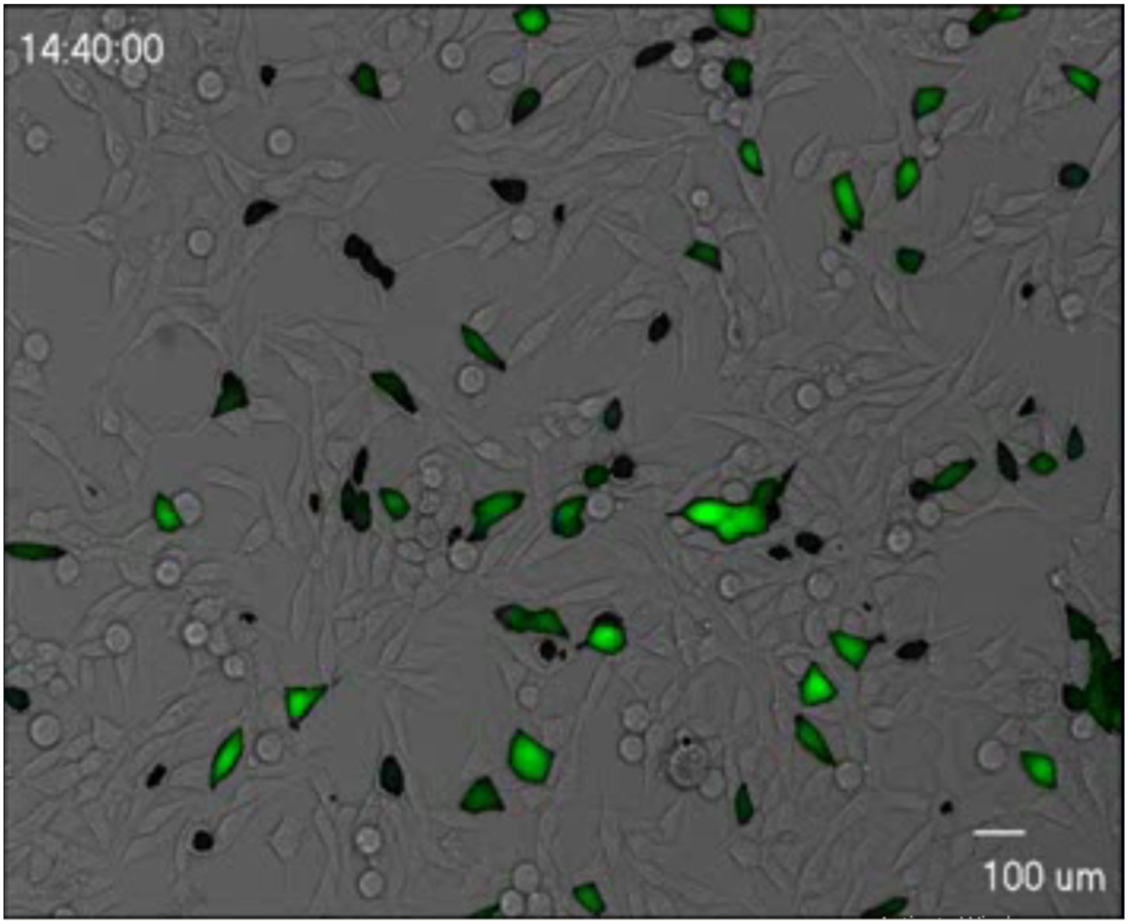

See Supplementary File 2

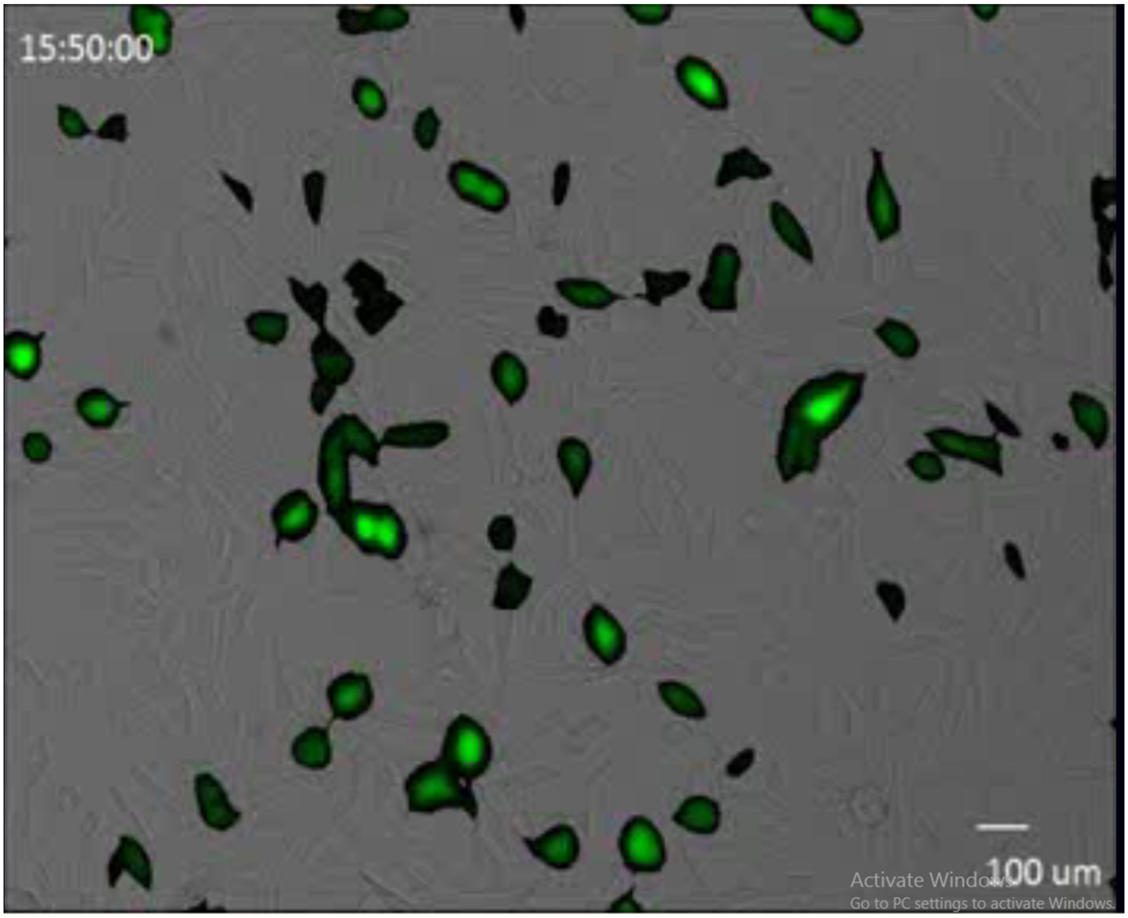

See Supplementary File 3

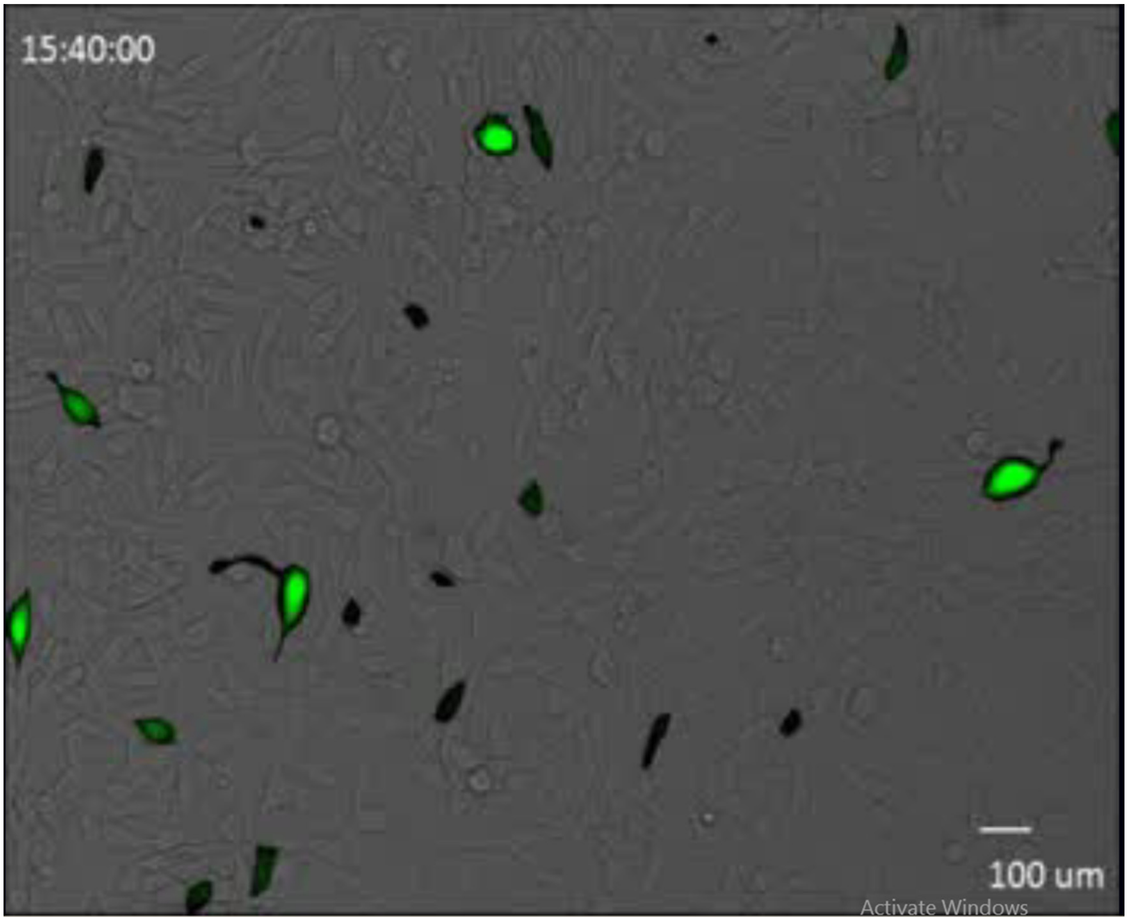

See Supplementary File 4

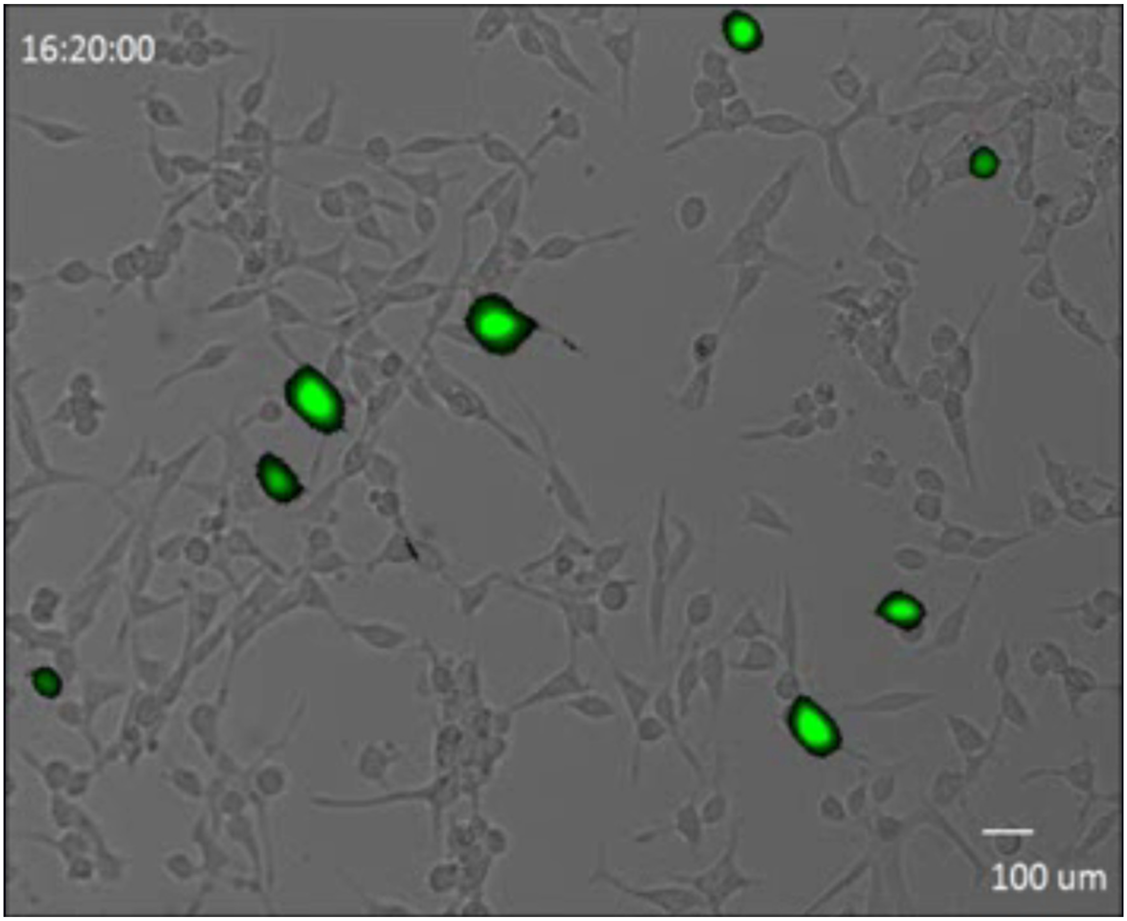

See Supplementary File 5

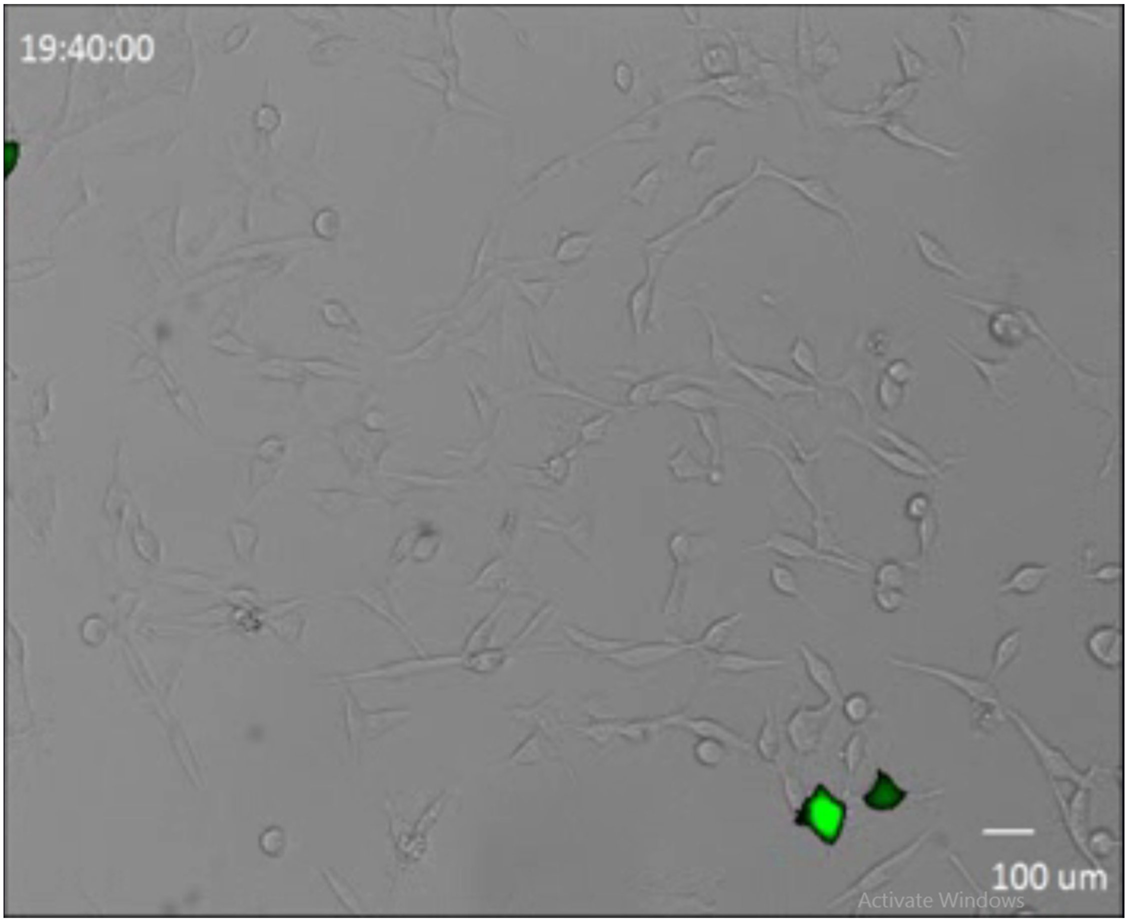

See Supplementary File 6

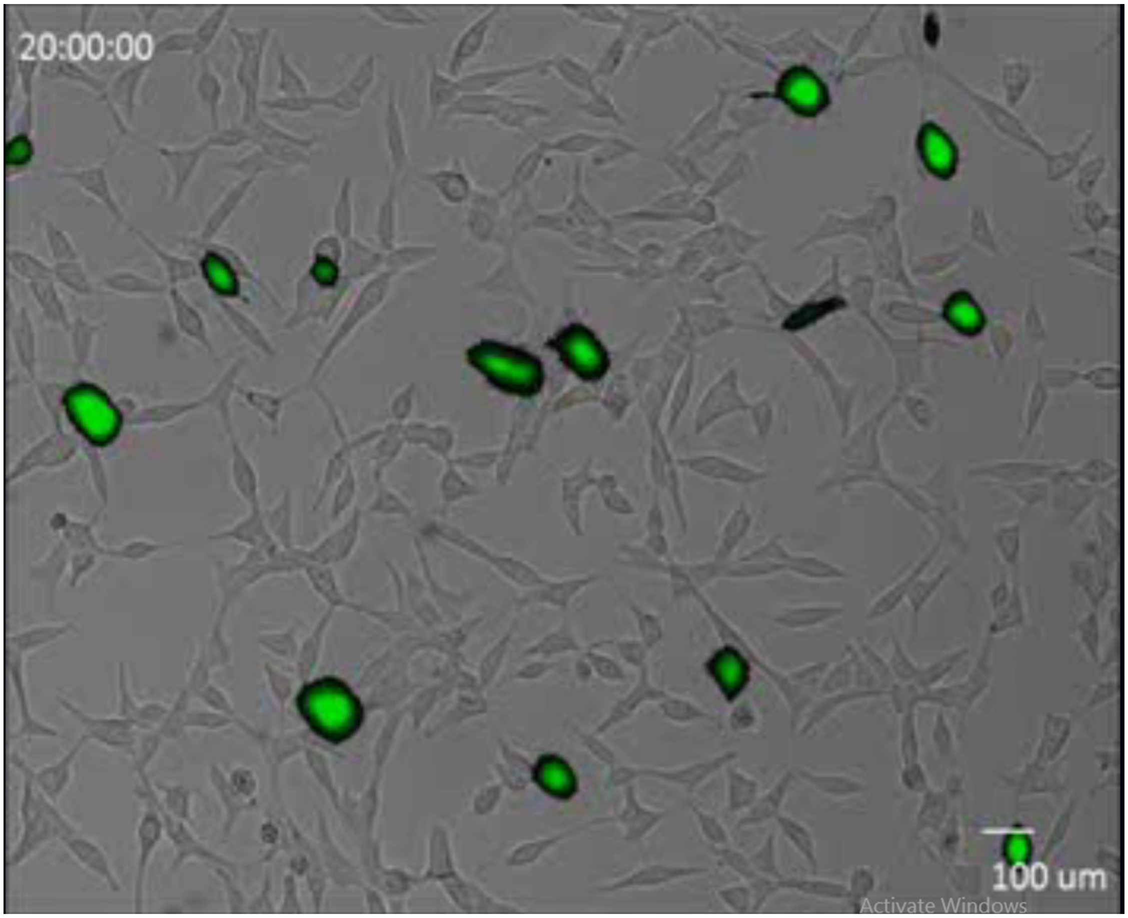

See Supplementary File 7

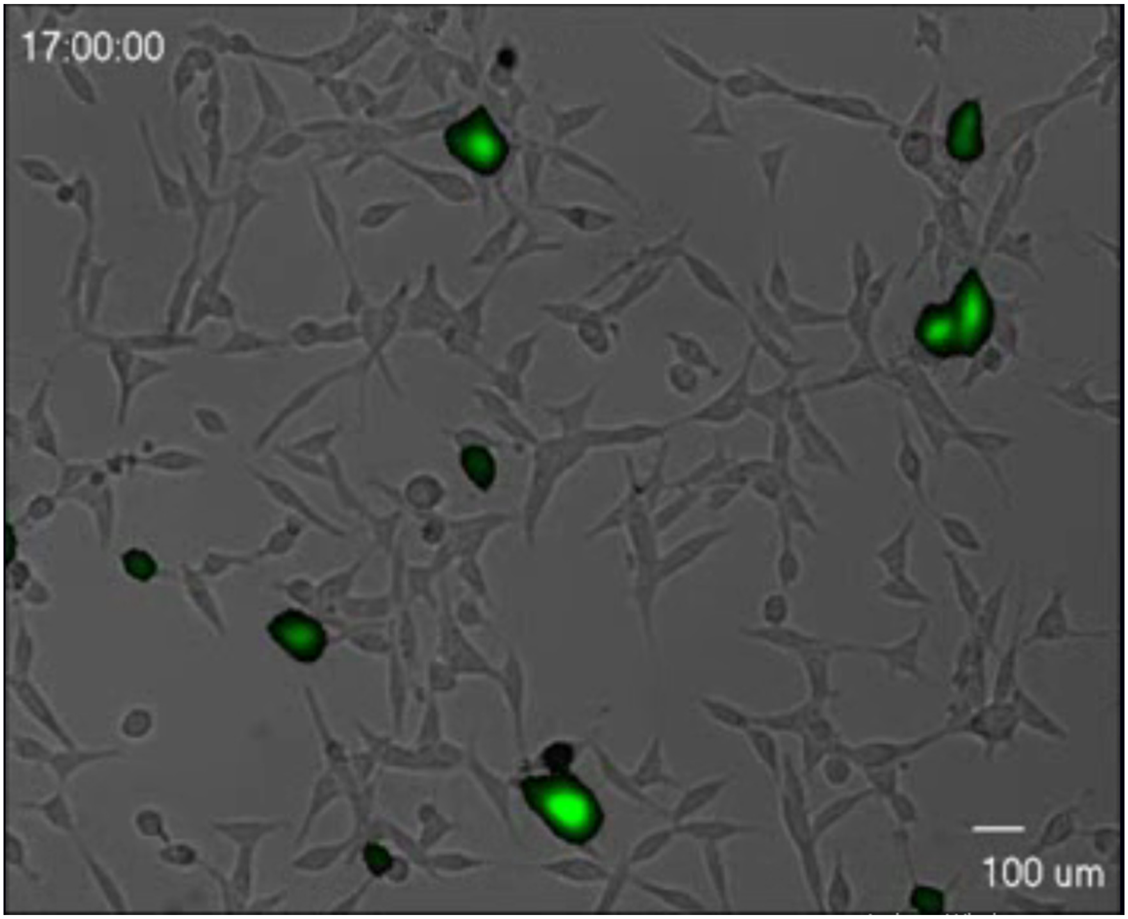

See Supplementary File 8

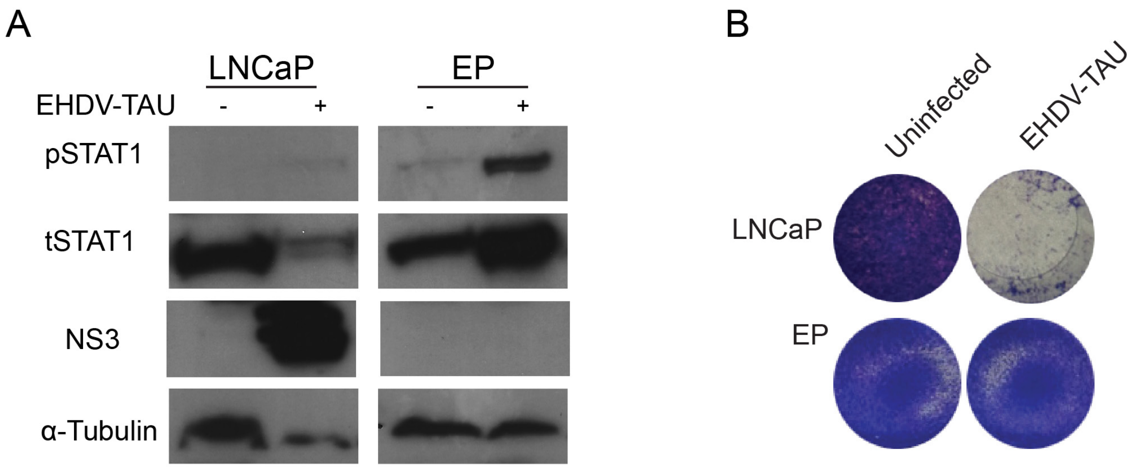

Supplementary Figure S1: EHDV-TAU infection in EP and LNCaP cells. A. Immunoblot analysis of EHDV-TAU infection of EP and LNCaP cells. Lysates (100 µg protein) of EP or LNCaP cells, infected (0.05 pfu/ml, 45 hpi) or not with EHDV-TAU were separated by 10 % SDS-PAGE, blotted on nitrocellulose and probed with antibodies against the indicated proteins (pSTAT1, tSTAT1, NS3 and α-tubulin). **B.** Crystal violet assessment of EHDV-TAU-induced death of cell cultures. LNCaP and EP cells were plated (70,000 cells/well) in 24 well plates. 24 h post platting, cells were infected with EHDV-TAU (0.05 pfu/ml, 45 h). At 45 hpi cells were fixed and stained with crystal violet. Panel depicts wells of a typical experiment.

Supplementary Table S1: List of primers used in this study

See Supplementary File 9

Supplementary Table S2: List of NFkB target ISGs. List of NFkB target genes, obtained from http://www.bu.edu/nf-kb/gene-resources/target-genes/ were crossed with a list of 500 ISGs [43]. The resulting 51 genes are common to both lists

See Supplementary File 10
